# Biogeography of acoustic biodiversity of NW Mediterranean coralligenous reefs

**DOI:** 10.1038/s41598-021-96378-5

**Published:** 2021-08-20

**Authors:** Lucia Di Iorio, Manon Audax, Julie Deter, Florian Holon, Julie Lossent, Cédric Gervaise, Pierre Boissery

**Affiliations:** 1 Chorus Institute, 38100 Grenoble, France; 2 Andromède Océanologie, 34130 Mauguio, France; 3 MARBEC, CNRS, IFREMER, IRD, Université de Montpellier, 34095 Montpellier, France; 4 Agence de l’Eau Rhône Méditerranée Corse, 13001 Marseille, France

**Keywords:** Behavioural ecology, Biodiversity, Biogeography, Community ecology, Conservation biology, Ecology

## Abstract

Monitoring the biodiversity of key habitats and understanding the drivers across spatial scales is essential for preserving ecosystem functions and associated services. Coralligenous reefs are threatened marine biodiversity hotspots that are challenging to monitor. As fish sounds reflect biodiversity in other habitats, we unveiled the biogeography of coralligenous reef sounds across the north-western Mediterranean using data from 27 sites covering 2000 km and 3 regions over a 3-year period. We assessed how acoustic biodiversity is related to habitat parameters and environmental status. We identified 28 putative fish sound types, which is up to four times as many as recorded in other Mediterranean habitats. 40% of these sounds are not found in other coastal habitats, thus strongly related to coralligenous reefs. Acoustic diversity differed between geographical regions. Ubiquitous sound types were identified, including sounds from top-predator species and others that were more specifically related to the presence of ecosystem engineers (red coral, gorgonians), which are key players in maintaining habitat function. The main determinants of acoustic community composition were depth and percentage coverage of coralligenous outcrops, suggesting that fish-related acoustic communities exhibit bathymetric stratification and are related to benthic reef assemblages. Multivariate analysis also revealed that acoustic communities can reflect different environmental states. This study presents the first large-scale map of acoustic fish biodiversity providing insights into the ichthyofauna that is otherwise difficult to assess because of reduced diving times. It also highlights the potential of passive acoustics in providing new aspects of the correlates of biogeographical patterns of this emblematic habitat relevant for monitoring and conservation.

## Introduction

Marine biodiversity plays a key role in maintaining ecosystem functions and providing numerous services^1,2^. Monitoring and tracking biodiversity at large spatial scales is challenging, but is urgently needed to build understanding of the drivers of biodiversity and support management and conservation, particularly in light of the vulnerability and rapid degradation of certain marine ecosystems^3,4^.

In the Mediterranean Sea, coralligenous reefs are emblematic coastal habitats that constitute a hotspot of biodiversity, hosting approximately 20% of the Mediterranean species^5^ despite covering only 0.1% of total surface area (2760 km^2^)^6^. Their richness, biodiversity, biomass and productivity are considered equivalent to those of tropical coral reef assemblages^7^. Coralligenous reefs can be found between 20 and 120 m depth and are composed of a hard substrate formed by concretion-forming organisms and the associated fixed biota that dwells in dim light conditions^8^. Several endangered species thrive in coralligenous communities, such as the Mediterranean red coral (*Corallium rubrum*) and the red gorgonian (*Paramuricea clavata*)^9^. Coralligenous reefs provide habitats, feeding grounds, recruitment and nursery sites for a myriad of fish species^9,10^ and are also fisheries hotspots^11^. These habitats are, however, highly vulnerable and exposed to numerous anthropogenic disturbances^12–15^, as well as climate change^16^. Consequently, coralligenous reefs are considered as priority conservation zones (Habitats Directive 92/43/CCE, Protocol for Special Protected Areas UNEP-MPA-RAC/SPA, 2008, Marine Strategy Framework Directive 2008/56/EC).

Monitoring biodiversity is particularly difficult in coralligenous reefs because they are spatially heterogeneous and less accessible than other coastal habitats (e.g., *Posidonia oceanica* meadows)^17^. Most monitoring methods are based on visual observations^18–21^ that tend to be episodic and diurnal, require complex diving logistics, and focus on benthic assemblages. Fish communities associated to coralligenous reefs are still poorly described, particularly at great depths, where diving times are reduced^9,22^. Recording the sounds present in habitats is an innovative and effective way of acquiring quantitative information on biodiversity at relevant temporal and spatial scales, and this non-invasively and irrespective of water turbidity, temperature, or depth^23,24^. Fish sounds are a major source of ambient noise in coastal environments^25,26^. Sound is used for communication by many fish species^27,28^, forming acoustic communities that show a clear link to taxonomic diversity^29,30^. Fish sounds therefore have good potential as proxies for biodiversity^24^. Furthermore, communication is a behaviour that can be rapidly tuned to habitat conditions^31,32^. Consequently, modifications in acoustic signals and variability in acoustic diversity can be indicative of community composition and ecological state^32,33^.

Despite the potential of fish sounds for assessing biodiversity and the key ecological role of coralligenous reefs, to date no passive acoustic studies have been conducted in this Mediterranean biodiversity hotspot. There is therefore a lack of knowledge on the environmental drivers of fish-related acoustic diversity and community composition. Biogeography, i.e., the distributional dynamics of taxa individually and collectively^34^, provides fundamental insights into the forces influencing the dynamics of biological diversity^35,36^. There is a paucity of studies on the geographical variation of biological sounds and potential links to habitat and the environment, particularly in the marine realm^37^. This is however necessary to explore the correlates of biogeographical patterns and establish how the environment affects community composition and biodiversity. In this study we report the fish-related acoustic biodiversity, biogeography, and community composition in coralligenous reefs across the north-western Mediterranean basin using passive acoustic monitoring (PAM) from 27 sampling sites covering almost 2000 km of coastline and depth ranges between 20 and 65 m. We applied community ecology principles to analyse the drivers shaping acoustic biodiversity of coralligenous reefs. This approach allows identification of habitat or range-restricted acoustic patterns, which can be used to assess the sensitivity of communities or species to environmental change.

## Results

### Biogeography and diversity of fish-related acoustic communities in coralligenous reefs

**Figure 1:**
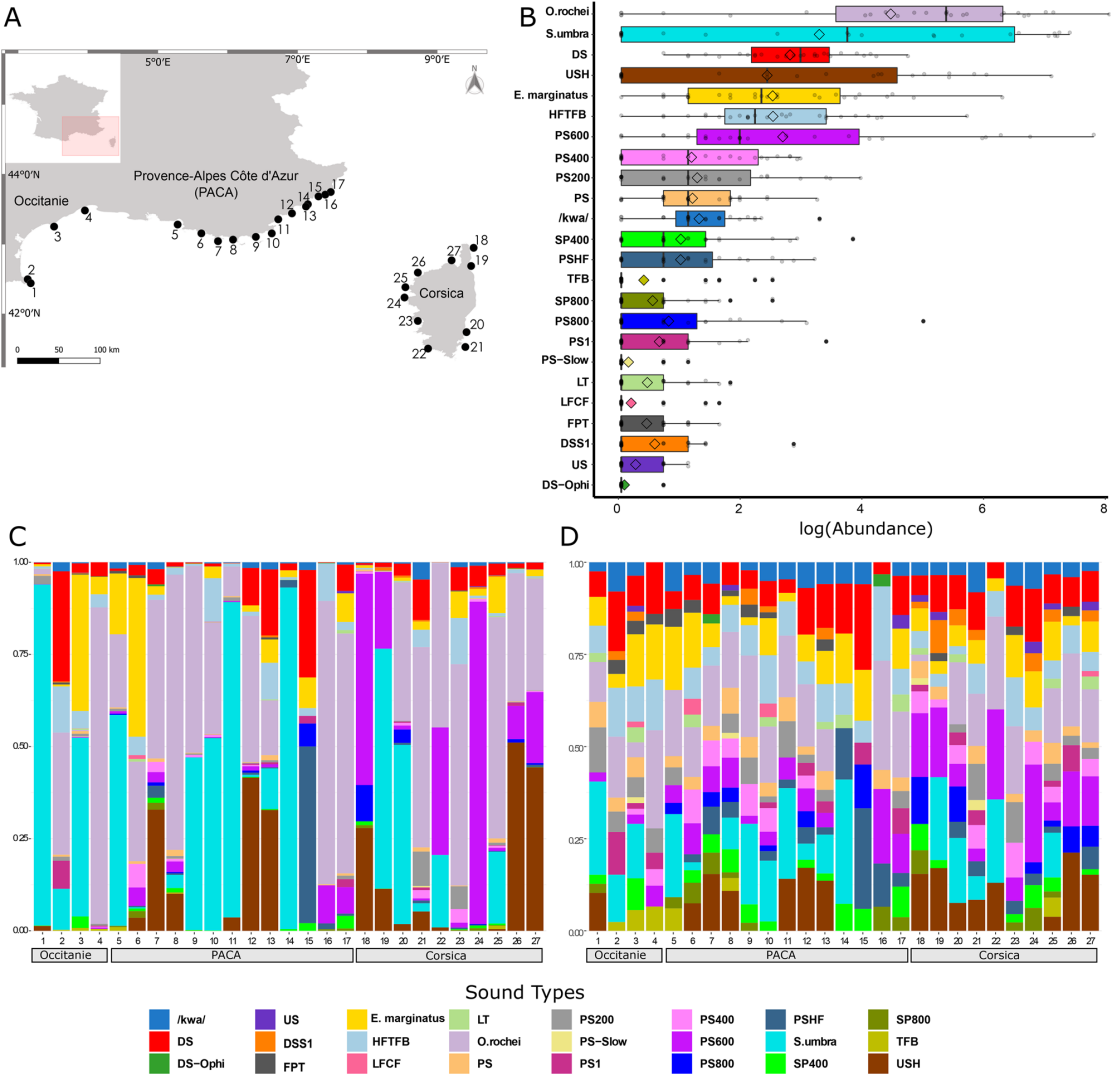
Biogeography of the acoustic biodiversity of coralligenous fish sounds. (**A**) Map of the 27 sampling sites of the CALME network across three areas of the north-western Mediterranean. Numbers indicate sites as listed in Supplementary Table S1. (**B**) Distribution of the total log relative abundance of each sound type (c.f. Supplementary Table S2) from all stations with the more abundant sound types on the top and the less abundant on the bottom. In each boxplot graph, diamonds indicate mean values (all stations) and grey dots indicate single values recorded from each station. (**C**) Bar chart showing the acoustic diversity of each site in different geographic regions based on the identified sound types (indicated by different colours) and their proportions of relative abundances. (**D**) Acoustic diversity of each site per geographic region based on the identified sound types (indicated by different colours) and the log of their occurrences to reduce the weight of abundant sounds and highlight “rare” sound types. Map 1A was created using QGIS.org, 2021. QGIS Geographic Information System. QGIS Association. http://www.qgis.org.

**Figure 2:**
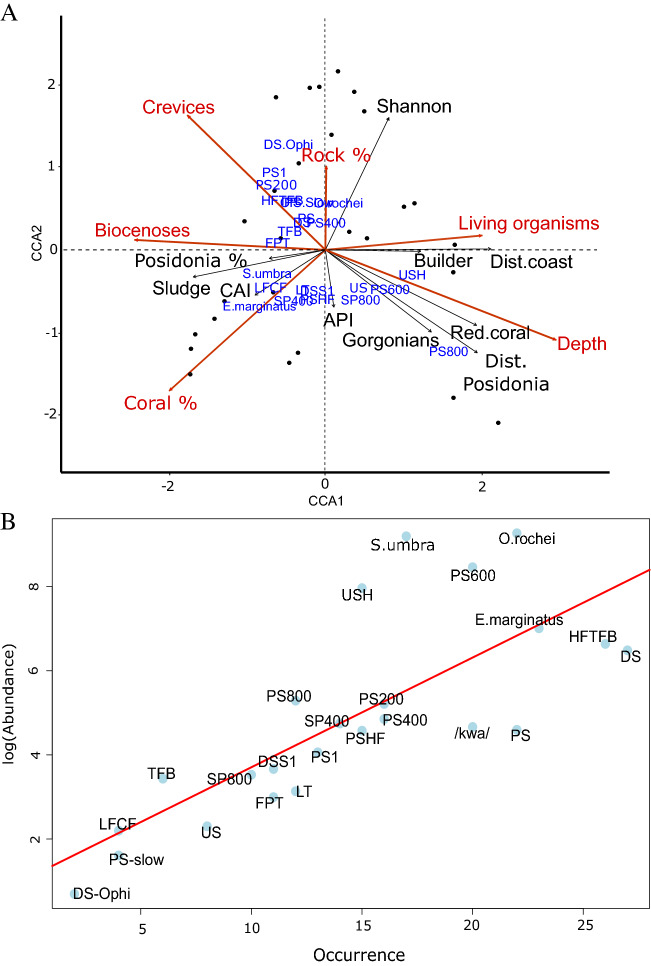
(**A**) Canonical correspondence analysis ordination plot of the acoustic community composition of coralligenous reefs based on Bray–Curtis dissimilarities of relative abundances of N = 24 sound types (blue) at 27 sampling sites (black dots) showing the influence of all environmental variables (arrows, c.f. Table 1), including the most relevant ones used for model testing (in red). Builder = Structuring species, Posidonia % = percent of *Posidonia oceanica*, Rock % = percent of rocky substrate, Coral %: percent of coralligenous outcrops, Dist. Posidonia = Distance from the closest *P. oceanica* meadow. (**B**) Occupancy-abundance plot showing the abundance of sound types across all sites. Sound types on the top right are those that are more common, while those on the bottom left occur only at a few sites (c.f., Supplementary Tables S2 and S3).

A total of 31,700 putative fish sound occurrences were recorded at the 27 sampling sites (Fig. 1A, Supplementary Table S1). Sound occurrences varied between 20 and 7946 with a mean value of 1344 (± 1465 SD) sound occurrences per site and 81 (± 77 SD) sound occurrences per hour of recording. Based on acoustic features a total of 28 sound types likely emitted by fish were identified, 24 of which were used for diversity analyses as some were merged to form the *Sciaena umbra* and *Epinephelus marginatus* categories (see methods, Supplementary Table S2). Sound type richness at sampling sites varied between 7 and 20 (14 ± 4 mean ± SD, n = 27 sites) (Fig. 1; Supplementary Table S3). Except for 4 sound types that could be attributed to known species or genera (*Ophidion rochei, S. umbra*, *E. marginatus, Scorpaena* spp.), all others were of unknown origin. Differences in relative sound abundances across sampling sites are mainly explained by the presence of sound types that are repeated in long series lasting up to several hours and therefore dominating acoustic communities. This was the case for 4 of the 7 most abundant sound types (Supplementary Table S3, Fig. 1B): *O. rochei*^38^, *S. umbra*^39^, USH, which is an upsweeping sound repeated in a series of 4–5 signals, and PS600, a series of pulses with a peak frequency around 600 Hz (Supplementary Fig. S1). Among sound types that are not repeated in long series, *E. marginatus*, the downsweeping DS sound type and the HFTFB sound type (c.f. Supplementary Table S2) were the most abundant (Supplementary Table S3, Figs. 1B and 2B). The occurrence of these 7 abundant sound types did, however, differ across sampling sites and regions. PS600 was generally widespread, occurring at 74% of the recorded sites, but was most abundant in Corsica (Fig. 1B,C, Supplementary Tables S4–S6). The USH sound type occurred at 55% of sampling sites, and although mainly present in Corsica and Provence-Alpes-Côte d'Azur (PACA), it was significantly more abundant in Corsica (Fig. 1C,D, Supplementary Tables S4–S6). *Ophidion rochei, E. marginatus,* DS and HFTFB sound types occurred at over 80% of sampling sites, but did not exhibit particular regional patterns (Fig. 1C,D, Supplementary Tables S4–S6). The relationship between sound type abundances and occurrences (i.e., presence across sampling sites) is illustrated in Fig. 2B. The DS sound and HFTFB were the most widespread sound types (occurred at over 26 sites) followed by *E. marginatus*, *O. rochei* and PS sounds (present at over 23 sites). These 5 sound types can therefore be considered as ubiquitous or “generalists” across Mediterranean coralligenous reefs. *Sciaena umbra* and USH sounds, although highly abundant, only occurred at around 15 of the 27 sites. The rarest and most site-specific sound types were DS-Ophi, LFCF and PS-slow (Fig. 2B, Supplementary Table S3).

### Environmental drivers of acoustic composition

Habitat and geographical variables (Table 1) were used to assess drivers of acoustic diversity. According to the results of the Canonical Correspondence Analysis (CCA), sound type composition was significantly influenced by depth, percentage surface area of coralligenous reefs, the number of biocenoses, and to a lesser extent the crevice percent cover and the percent of rocky reefs (Table 2). Inspection of the CCA ordination plot (Fig. 2A) allows association of sound types to habitat or geographical variables. For instance, the occurrence of the USH, PS600, PS800 and SP800 sound types appeared to be associated to variables characterizing coralligenous reefs, such as the presence of structuring species, red coral (*C. rubrum*) and gorgonians (Fig. 2A), while *E. marginatus*, *O. rochei* and *S. umbra* sound types were weakly or not related to these key habitat variables.

**Table 1:** List of geographical and habitat variables tested as drivers of coralligenous acoustic fish communities extracted from the Medtrix platform (www.medtrix.fr, RMC Water Agency/Andromède Océanologie).

Geographical variables	Habitat variables
Distance from the coast (m)	**Crevice percent cover (cm, dm, m)**
Distance from the closest *Posidonia oceanica* meadow (m)	Species percent cover
**Percent of coralligenous outcrops within a radius of 100 m (m**^**2**^**)**	**Percent of living fixed organisms**
**Percent of rocky substrate within a radius of 100 m (m**^**2**^**)**	Sediment percent cover
**Number of biocenoses**	Percent cover of gorgonians
Recording depth (m)*	Percent cover of red coral
Temperature at recording depth (°C)*	Coralligenous Assemblage Index, CAI (Deter et al.^22^) based on the Bryozoa percent cover, sediment percent cover, builder species percent cover
	Percent cover of structuring species
	Shannon Index of fixed species
	Simpson Index of fixed species

Descriptions of the habitat variables from the field stations are available in Deter et al. 2012. Variables selected by the model-building process for the canonical correspondence analysis are highlighted in bold.

*Environmental rather than geographical variable.

**Table 2:** Results of Canonical correspondence analysis testing for a link between acoustic communities and environmental variables (N = 27).

Variable	Df	F	p
Depth	1	5.04	0.001***
Percent of coralligenous reefs	1	3.45	0.002**
Number of biocenoses	1	2.63	0.01**
Crevice percent cover	1	2.17	0.042*
Percent of rocky reefs	1	2.22	0.036*
Percent living organisms	1	1.81	0.069

Only relevant metrics resulting from the stepwise variable selection are reported.

***p < 0.001, **p < 0.01, *p < 0.05.

Permutational multivariate analysis of variance (PERMANOVA) testing for the effect of categorical variables (environmental status, morphology) on β-diversity revealed that covariate depth had the strongest effect on acoustic communities (Table 3). Environmental status also significantly influenced acoustic community composition, while reef morphology had no significant effect (Supplementary Figs. S2 and S3, Table 3). The similarity percentage analysis (SIMPER) revealed that PS400 was the most discriminating sound type between environmental status conditions, with relative abundances being significantly higher in reefs considered in good environmental condition compared to those in poor environmental condition (Supplementary Table S7, Fig. S3). Sound type richness was greater in good vesus poor environmental condition, but differences were not significant (environmental status: F = 2.06, *p* = 0.15, Supplementary Fig. S4). However, this result is likely affected by one site considered as in good condition but exhibiting low sound type richness (Supplementary Fig. S4, site 22 Murtoli, Corsica in Supplementary Table S1).

**Table 3:** Results of the PERMANOVAs test for the influence of the fixed components environmental state and reef morphology on acoustic communities.

Variable	Df	*pseudo* F	*p*
State	1	2.26382	0.035*
Morphology	1	0.47001	0.859
Depth	1	2.89688	0.008**
State*morphology	1	2.07306	0.039*

The PERMANOVAs was based on log-standardized relative sound abundances (N = 24 sound types) and on Bray–Curtis dissimilarities (N = 27 sites).

*p < 0.05; *p* values were obtained using 9999 permutations.

Finally, acoustic communities of sites recorded in distinctive years did not statistically differ either at the community level (N = 28, F = 0.7, *p* = 0.71) or in sound type richness (N = 28, F = 0.24, *p* = 0.63). Moon phase had no significant effect on acoustic community composition (N = 28, F = 0.72, *p* = 0.77). These results indicate that overall acoustic communities did not significantly change between years, although differences exist in relative abundances or at specific sites (Supplementary Fig. S5).

## Discussion

This study generated an unprecedentedly large-scale georeferenced map of acoustic biodiversity of the second most important biodiversity hotspot of the north-western Mediterranean Sea. Despite the ecological importance and conservation value of coralligenous reefs, as well as the potential of PAM for monitoring biodiversity in marine ecosystems, this ecosystem had not previously been acoustically characterized. This basin-wide study reveals that acoustic communities of coralligenous reefs are diverse, composed of at least 28 fish sound type categories. This is over four times as many as observed in other Mediterranean habitats such as non-coralligenous rocky reefs or seagrass meadows^30,40^, equivalent or somewhat less than coral reef fish acoustic diversity^41,42^. Coralligenous reefs therefore host the highest acoustic biodiversity reported so far in the Mediterranean Sea. Acoustic communities varied across sites in terms of sound type composition, richness (14 ± 4 sound types), and abundance (1344 ± 1465 sounds). Coralligenous reefs represent complex habitats that are characterized by high structural heterogeneity and the development of several different benthic communities^9,43^. Variations in fish-related acoustic diversity may be linked to this habitat variability.

The biogeography of fish-related acoustic communities revealed regional variation in sound type composition. Although sharing similarities with PACA (Supplementary Fig. S2), acoustic biodiversity in the Occitanie region differed from that of the two other regions, with for instance the upsweeping sound with harmonics (USH) and the pules series centred around 600 Hz (PS600) being poorly represented (Fig. 1). USH and PS600, as well as PS800 and DSS1, were the sound types that mostly differentiated Corsica from PACA. Regional differences in diversity measures of coralligenous benthic assemblages have been reported along French Mediterranean coasts^44,45^, which may partly explain the differences in acoustic composition observed here.

Sound types varied in terms of relative abundance and occurrence at the study sites. Downsweeping sounds (DS) occurred at all coralligenous reefs, but the most abundant and ubiquitous (“generalist”) sound types were the *E. marginatus* and *O. rochei* sounds. Other abundant sound types that dominated acoustic communities locally at given sites and globally across the three regions were the *S. umbra*, PS600 and USH sound types. Of these more ubiquitous and/or abundant sound types, only PS600 and USH appear to be specific to coralligenous reefs. In fact, 60% of the sound repertoire identified in this study is known to also occur on rocky reefs^30^. This indicates the existence of an overlap of coralligenous reef with rocky reef acoustic biodiversity, which is not surprising considering that coralligenous algae mainly grow on rocky substrates^46^. 40% of the sound types described appear to be, so far, strongly related to coralligenous reefs. Of these, the highly abundant USH and ubiquitous PS600 types, as well as the stereotyped pulse sequence PS800 are linked to habitat variables such as structuring species, red coral (*C. rubrum*) and gorgonians, which are key players in maintaining habitat functions^47,48^. Validation of associations of this kind are relevant for habitat monitoring and conservation. However, despite their high specificity and occurrence, the species emitting these sounds remain unknown. The USH sound type shares strong acoustic similarities with a sound recently described in a canyon at around 100 m depth and referred to as STFRP (Stereotyped Trains of Fast Repeated Pulses)^49^. Although only reported once until today, it suggests that this sound type is likely emitted by a fish species inhabiting deeper environments^49^.

Monitoring fish sounds as conducted here also allows indirect acquisition of information on the rarely assessed, more vagile ichthyofauna associated to coralligenous habitats. It is interesting to note that the number of acoustic sound types identified here (i.e., 28) is almost equivalent to the number of fish species (between 30 and 40 species) reported from coralligenous reefs in a taxonomic survey^10^, which further supports the use of sounds as biodiversity indicators of this habitat. However, distinct sound types do not necessarily represent distinct species. Some fish species are known to produce more than one sound type^50,51^, and call differences may also be related to sex and age^52,53^. From the identified species dwelling on coralligenous reefs, only 6 are so far known to emit or be able to produce sounds: *Gobius cruentatus*, *Chromis chromis*, *Sciaena umbra, Epinephelus marginatus, Zeus faver, Scorpaena porcus and Scorpaena scrofa*^39,50,51,54–56^. Two of these species, *Sciaena umbra* and *Epinephelus marginatus*, are classified as vulnerable or endangered and rely on acoustic communication for reproduction^39,51^. The sounds of these species were among the most abundant sounds recorded in this study, suggesting sustained courtship behaviour in coralligenous reefs and a functional aspect of this habitat relevant for ecosystem resilience. Moreover, *E. marginatus* is a high-level predator and thus a functionally relevant species.

The main drivers of coralligenous acoustic community composition were depth (varying from 20 to 65 m) and the percentage surface area of coralligenous reefs in the recording area. This suggests that acoustic communities are both habitat-specific and depth-dependent. Vertical stratification patterns are known for species distribution^57,58^, but also acoustic diversity, as shown in forest habitats^59,60^. Fish fauna from the coralligenous community includes many species that inhabit a wide bathymetric range^9^, and vertical zonation of fish has been described for coral reefs^58,61^. In coralligenous reefs, previous studies based on visual and photographic data collection showed a depth-dependent distribution of benthic assemblages at the same sites as the present study^21,44,62^. Consequently, this depth-related variation of benthic communities likely influences fish assemblages related to the habitat and is a plausible explanation for the vertical zonation found in fish-related acoustic communities.

Environmental status also significantly determined acoustic communities, suggesting that fish assemblages are affected by the status of the fixed fauna of coralligenous reefs and that changes in fish acoustic communities may reflect habitat condition. Sites classified as being in good ecological status were those with relatively high abundance of structuring species, red coral and gorgonians and high percent of living organisms, all indicators of high biodiversity. Coralligenous reefs in good ecological condition showed higher sound type richness and abundance of PS600, USH, PS800 and PS400 sound types, but differences between conditions were only significant for PS400. The PS400 sound type may therefore be a pertinent indicator of environmental state in coralligenous reefs. Regional differences likely also played a role, as all sites in Corsica were considered to be in good environmental condition. Furthermore, compared to Corsica, dissimilarities among acoustic communities were lower in PACA and Occitanie (Supplementary Fig. S2). This may be a result of differences in human impact (reduced in Corsica), which is known to decrease species that are sensitive to disturbance and favour more generalist species, thus reducing β-diversity^44^. Moreover, as coralligenous reefs are acoustically rich, degradation of their acoustic environment by noise may impact fish communities. However, it remains to be verified whether the observed acoustic differences are linked to differences in anthropogenic pressures.

The identification of environmental drivers shaping marine acoustic communities at large scales is necessary to confirm the pertinence of PAM-based surveys to monitor and detect responses to human and environmental pressures. This first description of the biogeography of marine sound diversity in the Mediterranean Sea demonstrates that eco-acoustic approaches provide a promising non-invasive tool for exploration of the drivers of large-scale biogeographical patterns. As sound production may vary over time, time-series measurements would be useful to confirm the influence of habitat and environmental drivers on acoustic biodiversity and would allow more accurate determination of the temporal variability of fish biophony, which is known to vary on a weekly, monthly and seasonal basis in other coastal habitats^24^. As recordings were obtained over a 1-month period within the same season (80% recoded in the first 3 weeks of June, 20% in the first week of July) seasonal effects were not tested in this study. In sites monitored in the same season over 2 years in our study, acoustic community composition did not show interannual variability, supporting observations that fixed coralligenous assemblages show little variation over time^22^. However, fish belong to the vagile fauna and long-term recordings are needed in the future to assess whether acoustic communities are indeed stable over time.

Finally, given the pivotal ecological role of coralligenous habitats in the Mediterranean Sea, their vulnerability, and the need for surveillance using complementary and innovative survey methods^17^, this study opens new perspectives for the study and survey of this emblematic habitat. We introduce the survey of acoustic community diversity as an additional, complementary facet of biodiversity analysis, showing that it can infer information that is indicative of status and reflect traits related ecosystem functioning, both relevant for habitat conservation. This study also highlights the suitability of acoustic biogeography in assessing biodiversity patterns of the vagile fauna associated to coralligenous reefs at different depth belts, including those less accessible by conventional observational methods. If applied as part of a large-scale strategy, at critical depths and over the long term, monitoring of acoustic biodiversity can help understand the spatial and temporal dynamics of processes impacting this threatened habitat and its biodiversity and thus support habitat management initiatives.

## Methods

### Acoustic sampling

Sound recordings were obtained from the CALME acoustic monitoring program along the French western Mediterranean coast^63^. Recordings from 27 different coralligenous reefs across three regions were used for this study (Fig. 1A). Recordings were obtained over 3 years (2016–2018) under low wind regimes (< 10 kn) and during early summer to avoid seasonal effects on sound production (Supplementary Table S1). Fourteen of the 27 sites were recorded in two distinctive years (Supplementary Table S1). Because of the considerable distances between the locations of the reefs (65 km ± 45 km, mean ± S.D.), recordings could not be conducted simultaneously. Data were acquired using a HTI-92-WB hydrophone (High Tech Inc., USA) with a sensitivity of − 155 dB re 1 V/μPa and flat frequency response from 2 Hz to 50 kHz connected to an EA-SDA14 compact autonomous recorder (RTSys®, France). The device, which acquired sounds continuously at a 78 kHz sampling rate and 24-bit resolution, was bottom-moored with the hydrophone 1 m from the seafloor. At each recording date, the recorder was deployed in the afternoon and recovered the next day. Recordings were made during the night because most temperate fish predominantly vocalise nocturnally^39,64^ and interference with anthropogenic noise is largely reduced compared to during the day.

### Geographical and habitat data

Geographical and habitat data were obtained from the cartography platform MEDTRIX (www.medtrix.fr, RMC Water Agency/Andromède Océanologie) that collates data from all French Mediterranean coastal surveillance networks managed by the RMC Water Agency. Geographical and biocenosis data were acquired from the DONIA EXPERT cartography program (https://medtrix.fr/portfolio_page/donia-expert/) using the SIG platform QGIS® (Supplementary Fig. S6). The variables extracted (within a 100 m-radius of the recording location) are listed in Table 1, and include the number of different biocenoses and the area covered by coralligenous reefs and non-coralligenous rock to assess the influence of adjacent habitats. Reef depth at which the recordings occurred and temperature at recording depth were included as they may influence sound production in fish^65^. Morphology of reef formations (i.e., bank vs. rim) was also used to test for differences in acoustic fish communities. Habitat data on the structure, status and diversity of the sampled coralligenous reef assemblages (Table 1) were obtained from the RECOR surveillance program (https://medtrix.fr/portfolio_page/recor/) that characterizes and monitors French coralligenous reefs^21^.

### Acoustic data processing and diversity

Since most fish vocalize and mainly hear in the low (below 2000 Hz) frequency range^27^, audio recordings were down sampled to 4 kHz. Recordings from 7 pm to 7 am were analysed. Audio files were split into 10-s bins that were converted into a sequence of 10-s spectrograms (FFT size 256, Kaiser window with 80% overlap) using a custom-built MATLAB® (version R2014b) interface that allowed identification of potential fish sounds. Fish sound classification was based on acoustic properties as proposed in the dichotomous framework of Desiderà and co-authors^30^ using five axes: (1) type (frequency-modulated/pulse/constant frequency/high-entropy time–frequency block), (2) peak frequency, (3) number of repetitions per call, (4) rhythm of repetition (no rhythm/constant/variable), (5) repetition speed. Whenever known, sounds were attributed to fish species, such as the previously described *Ophidion rochei* sound^38^ or the /kwa/ attributed to *Scorpaena* spp.^54^. Two sound types were attributed to the brown meagre, *Sciaena umbra* (I-calls and R-calls^39^), and four to the dusky grouper, *Epinephelus marginatus* (low-frequency pulse series, low-frequency downsweeps, low-frequency downsweep series, low-frequency pulses and downsweeps^51,66^ (Supplementary Fig. S1). The sound types of each of these two species were combined to form a *S. umbra* and *E. marginatus* sound category. The other sound type categories (referred to as sound types throughout the text) generally consisted of one sound type only or of multiple sound types with very similar features, but for which a more specific classification was not possible^30^. Within each 10-s spectrogram only the presence of different sound types was noted. Abundances are therefore relative abundances given by the presence of a sound type in a 10-s bin. Sound type selections were summarized in csv output files that were then used in R software (version 3.6.0, R Core Team 2019) for community analyses and to calculate acoustic richness at each site.

### Effect of geographical and habitat variables on acoustic composition

Sound type diversity and relative abundances per site and geographic region (i.e., Corsica, PACA, Occitanie) was visualized using bar charts. A permutational similarity percentage (SIMPER) analysis^67^ was performed on a Bray–Curtis dissimilarity matrix^68^ to assess which sound types contributed most to regional differences. Data were log-standardized prior to testing to avoid biases linked to abundant sound types. Ordination methods were applied to test relationships between sound type composition (i.e., acoustic composition) and environmental variables (i.e., pooled geographical and habitat variables. Because 14 of the 27 sites were sampled twice in distinctive years, mean values of the sound type occurrences were used for these analyses. A canonical correspondence analysis (CCA) was used to test the effect of these continuous variables (Table 2) on acoustic composition. The CCA is a direct gradient analysis used to find the best dispersion of species, here sound type scores, and to relate these to combinations of environmental variables^69^. A model-building process was used to reduce the number of explanatory variables and select the most effective CCA model. A forward stepwise variable selection method was applied that gradually adds significant variables based on the Akaike information criterion (AIC) to help determine which are most relevant for the model^70^. The environmental variables included after stepwise selections are highlighted in Table 1. A permutation test was used to assess significance and explore the effects of the selected variables. Analyses were performed on R software using the packages *vegan* (version 2.5-6), function *simper, cca*, *stats*, and function *add1*.

### Effect of habitat condition and reef morphology on acoustic communities

Before testing the effect of habitat condition on acoustic composition, ecological status categories were defined based on the 10 habitat variables listed in Table 1 (right panel), extracted from the RECOR program for each of the 27 sampling sites. These variables were used to carry out a principal component analysis (PCA) using the *FactoMineR* and *factoextra* packages to assess the relationship between the habitat variables, as well as between the variables and the sampling sites. Two broad “ecological status” categories were established from the PCA based on the types of variables and their weights, identifying coralligenous reefs in potentially “good condition” versus reefs in rather “poor condition”. On the one hand, coralligenous reefs in sites dominated by structuring species, gorgonians, red coral, living organisms, with a high CAI (coralligenous assemblage index) were included in the “good” category (Supplementary Fig. S7). On the other hand, sites with low values of these variables and a high sediment (mud) content were considered to be in “poor condition”^71^ (Supplementary Fig. S7).

To test whether acoustic communities of coralligenous reefs differ between environmental states and/or reef morphology (i.e., bank or rim), multivariate analyses of variance based on permutations of distance matrices (PERMANOVA)^72^ were performed after log-standardization of sound type data (abundances of N = 24 sound types recorded from N = 27 sampling sites) to reduce the influence of abundant sound types. Environmental status (two levels: “good” or “poor”) and reef morphology (two levels: bank or rim) were set as fixed factors and depth (continuous variable), latitude and/or longitude as a covariates allowing testing of the interaction between depth, regions and environmental status and reef morphology respectively. The Akaike Information Criterion reviled that the best model was the one testing for quality and morphology as fixed factors and depth as covariate. Different communities were compared using a Bray–Curtis distance^68^ that quantifies the dissimilarity between two sites based on counts at each site (considering that group dispersion between conditions is homogenous). Sound types contributing to significant differences between “good” and “poor” reefs were identified using permutational similarity percentage (SIMPER) analyses performed using the *decostand, betadisper, adonis* and *simper* functions from the R package *vegan.* Sound type richness per site was also estimated based on the established dictionary and tested against environmental status and reef morphology, using analysis of variance, as all assumptions were met.

Finally, since 14 sites were recorded in two distinctive years and moon phases may influence fish sound production, a PERMANOVA (using ‘sound types × sampling sites’ matrices, N = 24 sound types, N = 14 samples, function *adonis2*) was performed to test for the effect of year (fixed two levels: year 1/year 2) and moon phase (fixed four levels: full/new/wax/wane).

## Supplementary Information

Supplementary Information.
