# Supplementary material for: Biogeography of acoustic biodiversity of NW Mediterranean coralligenous reefs
Source: Sci Rep. 2021 Aug 20;11:16991. doi: 10.1038/s41598-021-96378-5 (PMC8379277; doi:10.1038/s41598-021-96378-5)
Supplement: Supplementary file 1 — Supplementary Information. [file 41598_2021_96378_MOESM1_ESM.docx]

**Supplementary Information from**

# Biogeography of acoustic biodiversity of NW Mediterranean coralligenous reefs

**Supplementary TABLES**

**Table S1**. Description of the sites sampled, in France. PACA = Provence Alpes Côte d’Azur. N° correspond to the numbers of the sites in Fig. 1

| **Recording** | **Region** | **N°** | **Site name** | **Morphology** | **Recording date** | **Recording depth (m)** |
| --- | --- | --- | --- | --- | --- | --- |
| 1 | Occitanie | 1 | Sec Joël | bank | 30/05/2018 | 35 |
| 2 |  | 2 | Banyuls-sur-mer | bank | 21/06/2016 | 43 |
| 3 |  | 3 | Agde | bank | 20/06/2016 | 22 |
| 4 |  | 3 | Agde | bank | 31/05/2018 | 22 |
| 5 |  | 4 | Grand Travers | bank | 01/06/2018 | 25 |
| 6 | PACA | 5 | Frioul | bank | 06/06/2018 | 40 |
| 7 |  | 6 | Ciotat | bank | 11/06/2018 | 55 |
| 8 |  | 7 | Sicié | rim | 03/06/2016 | 42 |
| 9 |  | 7 | Sicié | rim | 14/06/2018 | 45 |
| 10 |  | 8 | Giens | bank | 04/06/2016 | 35 |
| 11 |  | 9 | Bormes | rim | 05/06/2016 | 37 |
| 12 |  | 10 | Cap Lardier | bank | 06/06/2016 | 38 |
| 13 |  | 10 | Cap Lardier | bank | 17/06/2018 | 45 |
| 14 |  | 11 | Bonneau | bank | 08/06/2016 | 36 |
| 15 |  | 12 | Cap Roux | bank | 09/06/2016 | 60 |
| 16 |  | 12 | Cap Roux | bank | 20/06/2018 | 56 |
| 17 |  | 13 | Golfe Juan | rim | 10/06/2016 | 37 |
| 18 |  | 13 | Golfe Juan | rim | 21/06/2018 | 38 |
| 19 |  | 14 | Bacon | rim | 11/06/2016 | 36 |
| 20 |  | 15 | Tombants Américains | rim | 12/06/2016 | 43 |
| 21 |  | 15 | Tombants Américains | rim | 22/06/2018 | 48 |
| 22 |  | 16 | Eze | bank | 13/06/2016 | 45 |
| 23 |  | 17 | Cap Martin | bank | 15/06/2016 | 48 |
| 24 |  | 17 | Cap Martin | bank | 23/06/2018 | 50 |
| 25 |  | 18 | Maccinagio | bank | 02/06/2017 | 65 |
| 26 |  | 18 | Maccinagio | bank | 25/07/2018 | 65 |
| 27 |  | 19 | Bastia | bank | 31/05/2017 | 62 |
| 28 |  | 19 | Bastia | bank | 24/07/2018 | 63 |
| 29 | Corsica | 20 | Tarco | bank | 30/05/2017 | 52 |
| 30 |  | 20 | Tarco | bank | 21/07/2018 | 55 |
| 31 |  | 21 | Rondinara | bank | 29/05/2017 | 46 |
| 32 |  | 22 | Murtoli | bank | 20/07/2018 | 43 |
| 33 |  | 23 | Parata | bank | 12/06/2017 | 60 |
| 34 |  | 23 | Parata | bank | 19/07/2018 | 60 |
| 35 |  | 24 | Cappu Rosso | rim | 10/06/2017 | 56 |
| 36 |  | 24 | Cappu Rosso | rim | 29/07/2018 | 65 |
| 37 |  | 25 | Focolara | bank | 09/06/2017 | 60 |
| 38 |  | 25 | Focolara | bank | 28/07/2018 | 60 |
| 39 |  | 26 | Calvi | rim | 05/06/2017 | 55 |
| 40 |  | 27 | Agriates | rim | 03/06/2017 | 56 |
| 41 |  | 27 | Agriates | rim | 26/07/2018 | 60 |

**Table S2.** Description of the identified sound type categories of Mediterranean coralligenous reefs. Spectrographic representations in supplementary figure S1 and Desiderà et al. 2019 ^1^.

| **Sound type** | **Description** |
| --- | --- |
| /kwa/ | sound emitted by *Scorpaena sp*. (Di Iorio et al. 2018^2^, Bolgan et al. 2019^3^) |
| DS | down-sweeping sound |
| DS-Ophi | downsweeping pulse series sharing similarities with *Ophidion rochei* calls |
| DSS1 | stereotyped series of short (0.03s) down-sweeps |
| FPT | fast pulse train |
| E. marginatus | combines four sounds likely associated to groupers (Bertucci et al. 2015^4^, Desiderà et al. 2019^1^) |
| HFTFB | higher-frequency (> 800 Hz) time-frequency block characterized by high entropy |
| LFCF | low-frequency (<200Hz) constant frequency call |
| LT | long tonal sound |
| O.rochei | typical *Ophidion rochei* call (Kéver et al., 2016^5^) |
| PS | pulse series |
| PS1 | stereotyped pulse series with increasing pulse period |
| PS200 | pulse series with peak frequency around 200 Hz sharing similarities with *S. umbra* calls; average pulse duration: 0.08 s and pulse periods: 0.27 |
| PS400 | pulse series with peak frequency around 400 Hz with variable pulse periods |
| PS600 | pulse series with peak frequency around 600 Hz with irregular but rather long pulse periods ranging between 0.3 s and 1.4 s |
| PS800 | pulse series with peak frequency around 800 Hz, pulse durations of around 0.007 s and regular pulse periods (~1 s) |
| PSHF | pulse series of higher frequency 900-1000 Hz without a regular temporal pattern |
| PS-Slow | pulse series with long (>3 s ) pulse periods |
| S.umbra | typical *Sciaena umbra* call (R and I calls, Picciulin et al. 2012^6^) |
| SP400 | single pulse with peak frequency around 400 Hz but repeated over time |
| SP800 | single pulse with peak frequency around 800 Hz but repeated over time |
| TFB | time-frequency block with high entropy |
| US | Upsweeping sound |
| USH | upsweeping sound series with harmonics |

**References cited:**

1. Desiderà, E. *et al.* Acoustic fish communities: sound diversity of rocky habitats reflects fish species diversity and beyond? *Mar. Ecol. Prog. Ser.* **608**, 183–197 (2019).

2. Di Iorio, L. *et al.* ‘Posidonia meadows calling’: a ubiquitous fish sound with monitoring potential. *Remote Sens. Ecol. Conserv.* **4**, 248–263 (2018).

3. Bolgan, M. *et al.* Sea chordophones make the mysterious /Kwa/ sound: identification of the emitter of the dominant fish sound in Mediterranean seagrass meadows. *J. Exp. Biol.* **222**, jeb196931 (2019).

4. Bertucci, F., Lejeune, P., Payrot, J. & Parmentier, E. Sound production by dusky grouper Epinephelus marginatus at spawning aggregation sites. *J. Fish Biol.* **87**, 400–421 (2015).

5. Kéver, L., Lejeune, P., Michel, L. N. & Parmentier, E. Passive acoustic recording of *Ophidion rochei* calling activity in Calvi Bay (France). *Mar. Ecol.* **37**, 1315–1324 (2016).

6. Picciulin, M. *et al.* Diagnostics of noctural calls of Sciena umbra (L., fam. Sciaenidae) in a nearshore Mediterranean marine reserve. *Bioacoustics* 1–12 (2012). doi:10.1080/09524622.2012.727277

7. Desiderà, E. Reproductive behaviours of groupers (Epinephelidae) in the Tavolara-Punta Coda Cavallo Marine protected area (NW Mediterranean Sea). (2019).

**Table S3.** Summary statistics of the relative abundances of coralligenous reef sound types of all sites combined. Minimal values (min) indicate the smallest number of sounds recorded per sampling site, and maximal (max) values the highest number of sounds recorded per sampling site. sd = standard deviation. *S. umbra* comprises two sound types and *E. marginatus*, four sound types.

| **Sound type** | **min** | **max** | **median** | **mean** | **sd** |
| --- | --- | --- | --- | --- | --- |
| O.rochei | 0 | 3030 | 207 | 392.07 | 120.87 |
| S.umbra | 0 | 1583 | 40 | 364.59 | 104.41 |
| PS600 | 0 | 2349 | 6 | 174.85 | 92.02 |
| USH | 0 | 1173 | 10 | 106.33 | 45.99 |
| E.marginatus | 0 | 523 | 9 | 40.93 | 19.43 |
| HFTFB | 0 | 292 | 8 | 28.22 | 10.88 |
| DS | 1 | 110 | 18 | 24.26 | 4.77 |
| PS800 | 0 | 142 | 0 | 7.33 | 5.25 |
| PS200 | 0 | 50 | 2 | 6.78 | 2.28 |
| PS400 | 0 | 18 | 2 | 4.74 | 1.16 |
| SP400 | 0 | 44 | 1 | 4.26 | 1.75 |
| /kwa/ | 0 | 25 | 2 | 3.93 | 0.95 |
| PS | 0 | 24 | 2 | 3.67 | 0.96 |
| PSHF | 0 | 23 | 1 | 3.59 | 1.11 |
| PS1 | 0 | 28 | 0 | 2.15 | 1.06 |
| DSS1 | 0 | 16 | 0 | 1.44 | 0.60 |
| SP800 | 0 | 11 | 0 | 1.26 | 0.46 |
| TFB | 0 | 11 | 0 | 1.15 | 0.52 |
| LT | 0 | 5 | 0 | 0.85 | 0.26 |
| FPT | 0 | 4 | 0 | 0.74 | 0.20 |
| US | 0 | 2 | 0 | 0.37 | 0.12 |
| LFCF | 0 | 4 | 0 | 0.33 | 0.18 |
| PS-Slow | 0 | 2 | 0 | 0.19 | 0.09 |
| DS-Ophi | 0 | 1 | 0 | 0.07 | 0.05 |

Numbers in the sound-type names indicate centre frequencies. DS = downsweeping sound, DS-Ophi = Ophidion-like sound type with pulses having a downsweeping component, DSS1 = stereotyped series of downsweeping sounds, FPT = fast pulse train, HFTFB = high-frequency (> 800 Hz) time-frequency block, LT = low-frequency tonal sounds, PS = pulse series with number indicating peak frequencies, PS1 = stereotyped PS, PSHF = high-frequency (>800 Hz) pulse series, PS-Slow = pulse series with regular long (>3 s ) pulse periods, TFB = high-entropy time-frequency block, US = upsweeping sound, USH = upsweeping sound with harmonics, (c.f., supplementary table S2).

**Table S4**. SIMPER analysis identifying the contribution of each sound type (*c.f.* supplementary table S2) to the Bray Curtis dissimilarity metric between the geographical regions Corsica and PACA. Sound types showing significant differences between regions are highlighted in bold.

| **Sound type** | **average contribution %** | **average abundance "Corsica"** | **average abundance "PACA"** | **cumulative contribution %** |
| --- | --- | --- | --- | --- |
| S.umbra | 0.053636 | 5.3636 | 0.046453 | 1.1546 |
| **PS600** | **0.049619** | **4.9619** | **0.039392** | **1.2596** |
| **USH** | **0.047787** | **4.7787** | **0.037276** | **1.282** |
| O.rochei | 0.046424 | 4.6424 | 0.042574 | 1.0905 |
| E.marginatus | 0.024485 | 2.4485 | 0.017322 | 1.4135 |
| HFTFB | 0.022711 | 2.2711 | 0.017719 | 1.2817 |
| PS400 | 0.02185 | 2.185 | 0.016109 | 1.3564 |
| **PS800** | **0.021688** | **2.1688** | **0.021072** | **1.0292** |
| PS200 | 0.020662 | 2.0662 | 0.018635 | 1.1088 |
| PSHF | 0.019449 | 1.9449 | 0.017608 | 1.1046 |
| DS | 0.018827 | 1.8827 | 0.013669 | 1.3773 |
| SP400 | 0.015269 | 1.5269 | 0.012105 | 1.2614 |
| **DSS1** | **0.014803** | **1.4803** | **0.013817** | **1.0714** |
| PS | 0.014251 | 1.4251 | 0.01066 | 1.3369 |
| CF | 0.013193 | 1.3193 | 0.010814 | 1.22 |
| SP800 | 0.012157 | 1.2157 | 0.011578 | 1.05 |
| PS1 | 0.011284 | 1.1284 | 0.011328 | 0.9961 |
| LT | 0.0082 | 0.82 | 0.008134 | 1.008 |
| FPT | 0.00727 | 0.727 | 0.007052 | 1.0308 |
| US | 0.006475 | 0.6475 | 0.006515 | 0.9939 |
| TFB | 0.005959 | 0.5959 | 0.011196 | 0.5322 |
| LFCF | 0.003901 | 0.3901 | 0.007605 | 0.513 |
| PS-Slow | 0.003063 | 0.3063 | 0.005604 | 0.5466 |
| DS-Ophi | 0.001846 | 0.1846 | 0.004392 | 0.4203 |

**Table S5**. SIMPER analysis identifying the contribution of each sound type (*c.f.* supplementary table S2) to the Bray Curtis dissimilarity metric between the geographical regions Occitanie and PACA. Sound types showing significant differences between regions are highlighted in bold.

| **Sound type** | **average contribution %** | **average abundance "Occitanie"** | **average abundance "PACA"** | **cumulative contribution %** |
| --- | --- | --- | --- | --- |
| S.umbra | 0.057573 | 5.7573 | 0.047867 | 1.2028 |
| O.rochei | 0.041356 | 4.1356 | 0.035056 | 1.1797 |
| Grouper | 0.0337 | 3.37 | 0.023446 | 1.4374 |
| USH | 0.033504 | 3.3504 | 0.032305 | 1.0371 |
| PS.meagre | 0.027001 | 2.7001 | 0.019071 | 1.4158 |
| HFTFB | 0.026868 | 2.6868 | 0.020731 | 1.2961 |
| **DS** | **0.025226** | 2.5226 | **0.01834** | **1.3755** |
| PSHF | 0.025107 | 2.5107 | 0.022181 | 1.1319 |
| PS600 | 0.023222 | 2.3222 | 0.019153 | 1.2124 |
| **SP400** | **0.021879** | 2.1879 | **0.016504** | **1.3257** |
| **PS1** | **0.020741** | 2.0741 | **0.020978** | **0.9887** |
| **TFB** | **0.018558** | 1.8558 | **0.014115** | **1.3147** |
| PS400 | 0.017549 | 1.7549 | 0.015046 | 1.1663 |
| PS | 0.016917 | 1.6917 | 0.012742 | 1.3277 |
| CF | 0.015984 | 1.5984 | 0.011932 | 1.3396 |
| **FPT** | **0.011483** | 1.1483 | **0.008919** | **1.2874** |
| LT | 0.010633 | 1.0633 | 0.009567 | 1.1114 |
| SP800 | 0.010163 | 1.0163 | 0.010248 | 0.9917 |
| PS800 | 0.009217 | 0.9217 | 0.011484 | 0.8026 |
| DSS1 | 0.00792 | 0.792 | 0.007705 | 1.028 |
| CF.200 | 0.005105 | 0.5105 | 0.007801 | 0.6544 |
| DSMF | 0.003828 | 0.3828 | 0.0058 | 0.66 |
| PS.Slow | 0.002791 | 0.2791 | 0.004461 | 0.6256 |
| DS.Ophi | 0.001944 | 0.1944 | 0.004654 | 0.4178 |

**Table S6**. SIMPER analysis identifying the contribution of each sound type (*c.f.* supplementary table S2) to the Bray Curtis dissimilarity metric between the geographical regions Occitanie and Corsica. Sound types showing significant differences between regions are highlighted in bold.

| **Sound type** | **average contribution %** | **average abundance "Occitanie"** | **average abundance "Corsica"** | **cumulative contribution %** |
| --- | --- | --- | --- | --- |
| **PS600** | **0.056802** | **5.6802** | **0.038367** | **1.4805** |
| S.umbra | 0.054773 | 5.4773 | 0.04356 | 1.2574 |
| USH | 0.048794 | 4.8794 | 0.034062 | 1.4325 |
| O.rochei | 0.044065 | 4.4065 | 0.031045 | 1.4194 |
| Grouper | 0.028399 | 2.8399 | 0.021119 | 1.3447 |
| PS.meagre | 0.026133 | 2.6133 | 0.016029 | 1.6304 |
| HFTFB | 0.023138 | 2.3138 | 0.015239 | 1.5183 |
| PS800 | 0.02105 | 2.105 | 0.02418 | 0.8706 |
| PS400 | 0.019638 | 1.9638 | 0.012927 | 1.5192 |
| PS1 | 0.018834 | 1.8834 | 0.017969 | 1.0481 |
| SP400 | 0.01871 | 1.871 | 0.01718 | 1.0891 |
| **TFB** | **0.017736** | **1.7736** | **0.01316** | **1.3477** |
| DS | 0.017076 | 1.7076 | 0.013101 | 1.3035 |
| PS | 0.01565 | 1.565 | 0.010572 | 1.4802 |
| CF | 0.015624 | 1.5624 | 0.011337 | 1.3782 |
| DSS1 | 0.013026 | 1.3026 | 0.012194 | 1.0683 |
| PSHF | 0.012218 | 1.2218 | 0.012303 | 0.9931 |
| **FPT** | **0.0109** | **1.09** | **0.007513** | **1.4507** |
| LT | 0.009809 | 0.9809 | 0.008948 | 1.0962 |
| SP800 | 0.008798 | 0.8798 | 0.011337 | 0.776 |
| DSMF | 0.005838 | 0.5838 | 0.00594 | 0.9827 |
| PS.Slow | 0.003945 | 0.3945 | 0.005537 | 0.7125 |
| CF.200 | 0.002741 | 0.2741 | 0.004255 | 0.6441 |
| DS.Ophi | 0 | 0 | 0 | 0 |

**Table S7**. SIMPER analysis identifying the contribution of each sound type (*c.f.* supplementary table S2) to the Bray Curtis dissimilarity metric between “good” and “poor” environmental status. Sound types showing significant differences are highlighted in bold.

| **Sound type** | **average contribution %** | **average abundance "good"** | **average abundance "poor"** | **cumulative contribution %** |
| --- | --- | --- | --- | --- |
| S.umbra | 0.055499 | 5.5499 | 3.3223 | 4.21374 |
| USH | 0.046395 | 4.6395 | 3.166 | 1.51083 |
| PS600 | 0.045905 | 4.5905 | 3.7169 | 1.48569 |
| O.rochei | 0.043415 | 4.3415 | 4.8277 | 4.47604 |
| **PS400** | **0.024142** | 2.4142 | **1.6984** | **0.43706** |
| PS200 | 0.02329 | 2.329 | 1.2082 | 1.81001 |
| E.marginatus | 0.022513 | 2.2513 | 2.3876 | 2.31078 |
| HFTFB | 0.021882 | 2.1882 | 2.4083 | 2.39071 |
| DS | 0.019412 | 1.9412 | 2.946 | 2.60786 |
| PS800 | 0.019397 | 1.9397 | 1.2352 | 0.31061 |
| SP400 | 0.016598 | 1.6598 | 0.8653 | 0.93184 |
| PS1 | 0.015983 | 1.5983 | 0.6025 | 0.81817 |
| PSHF | 0.015643 | 1.5643 | 0.8307 | 0.79709 |
| PS | 0.01562 | 1.562 | 1.0847 | 1.25013 |
| CF | 0.014265 | 1.4265 | 1.4668 | 1.04638 |
| **DSS1** | **0.014188** | 1.4188 | **0.9201** | **0.08664** |
| **TFB** | **0.011393** | 1.1393 | **0.169** | **0.66229** |
| SP800 | 0.009228 | 0.9228 | 0.4577 | 0.39726 |
| LT | 0.008056 | 0.8056 | 0.3995 | 0.34657 |
| FPT | 0.007535 | 0.7535 | 0.2978 | 0.44794 |
| UF | 0.005944 | 0.5944 | 0.2978 | 0.22397 |
| PS-Slow | 0.002813 | 0.2813 | 0.1378 | 0.08664 |
| LFCF | 0.002452 | 0.2452 | 0.1771 | 0 |
| DS-Ophi | 0 | 0 | 0 | 0 |

**Supplementary FIGURES**

**
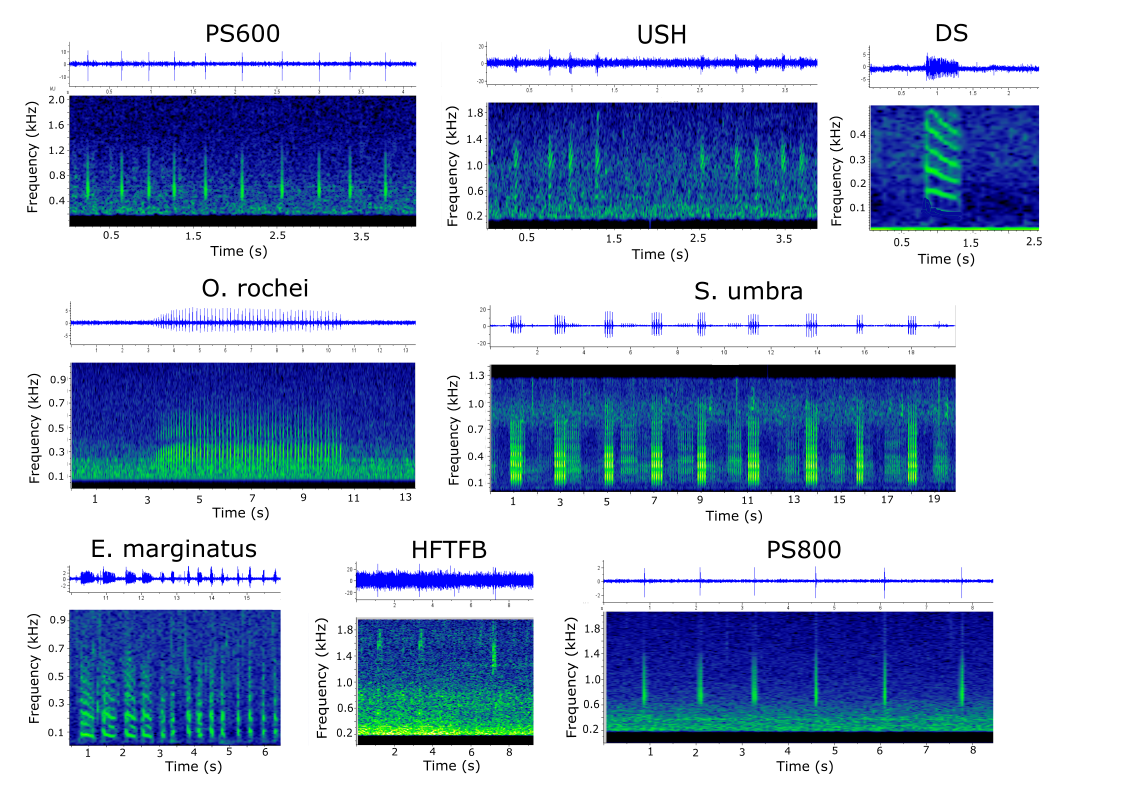
**

**Figure S1**. Waveforms and spectrograms of the most abundant sound types. The *S. umbra* sound type represents typical R-calls and the *E. marginatus* sound type a low-frequency downsweep and low-frequency pulse series (*c.f.,* supplementary table S2 & Desiderà 2019).


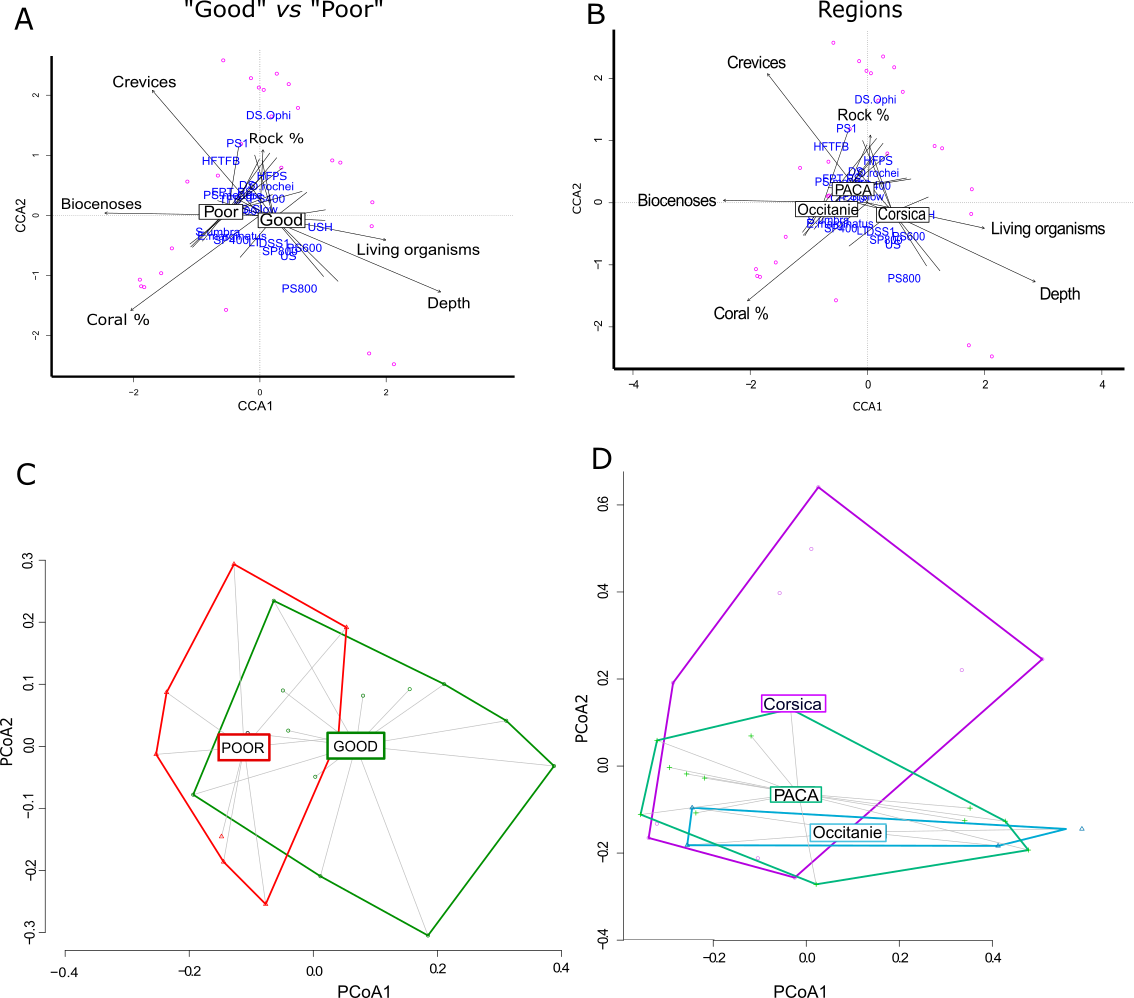


**Figure S2**. Influence of environmental status and geographic region on acoustic communities. **A & B**) Canonical correspondence analysis ordination plots of the acoustic community composition of coralligenous reefs based on Bray–Curtis dissimilarities of relative abundances of n=24 sound types (blue) in 27 sampling sites (black dots) showing the influence of the most relevant environmental variables (arrows) and adding the grouping variable environmental status (“good”, “poor”) or “region” (Occitanie, PACA, Corsica) (**B**). Coral %: percent of coralligenous outcrops. **C & D**) Principal coordinate analysis plots of the effect of environmental status (**C**) and geographical region (**D**) on fish-related acoustic communities based on Bray-Curtis’ distances.


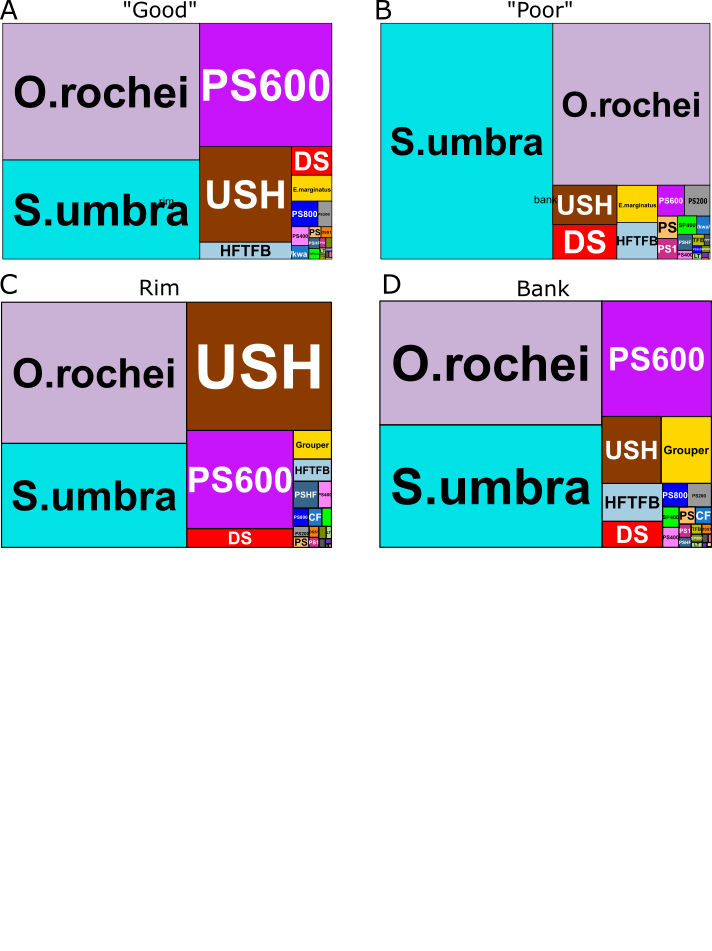


**Figure S3**. Influence of environmental status and morphology on acoustic communities. Treemaps showing relative occurrences of the different sound types (*c.f.,* supplementary table S2) from sites associated to “good” (**A**) and “poor” (**B**) environmental status, or from sites with different rock morphology: rim (**C**), bank (**D**).


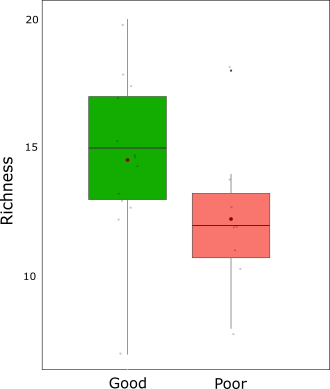


**Figure S4**. Boxplot of sound type richness in relation environmental status. Red dots indicate mean values and grey dots single measures.


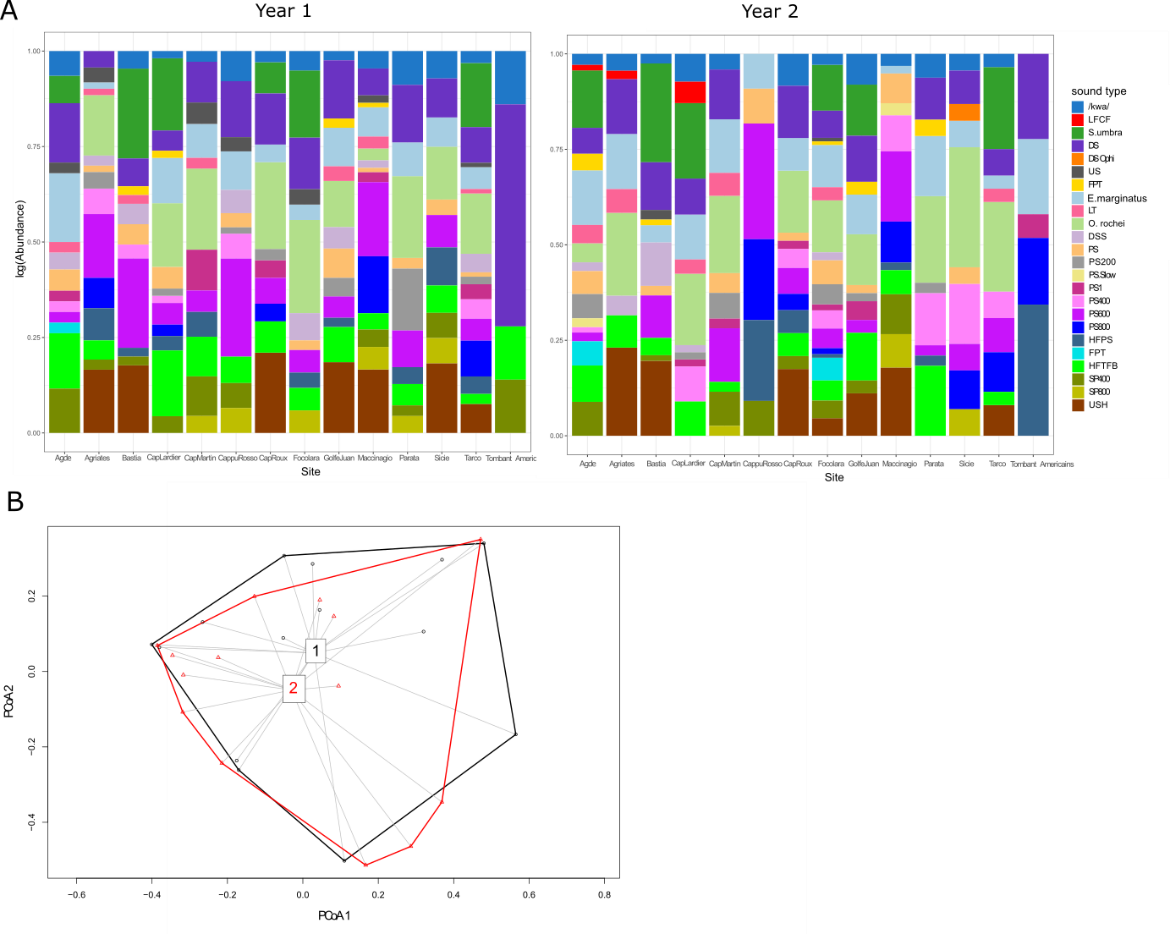


**Figure S5. A**) Bar chart showing the acoustic diversity of each site in two different years based on the identified sound types (indicated by different colours) and their relative log-standardized occurrences. **B**) Multivariate homogeneity of group variance for acoustic communities by year (1=year 1, 2= year 2) based on Bray-Curtis’ distances.

1 - Sec Joël 2 – Banyuls-sur-Mer


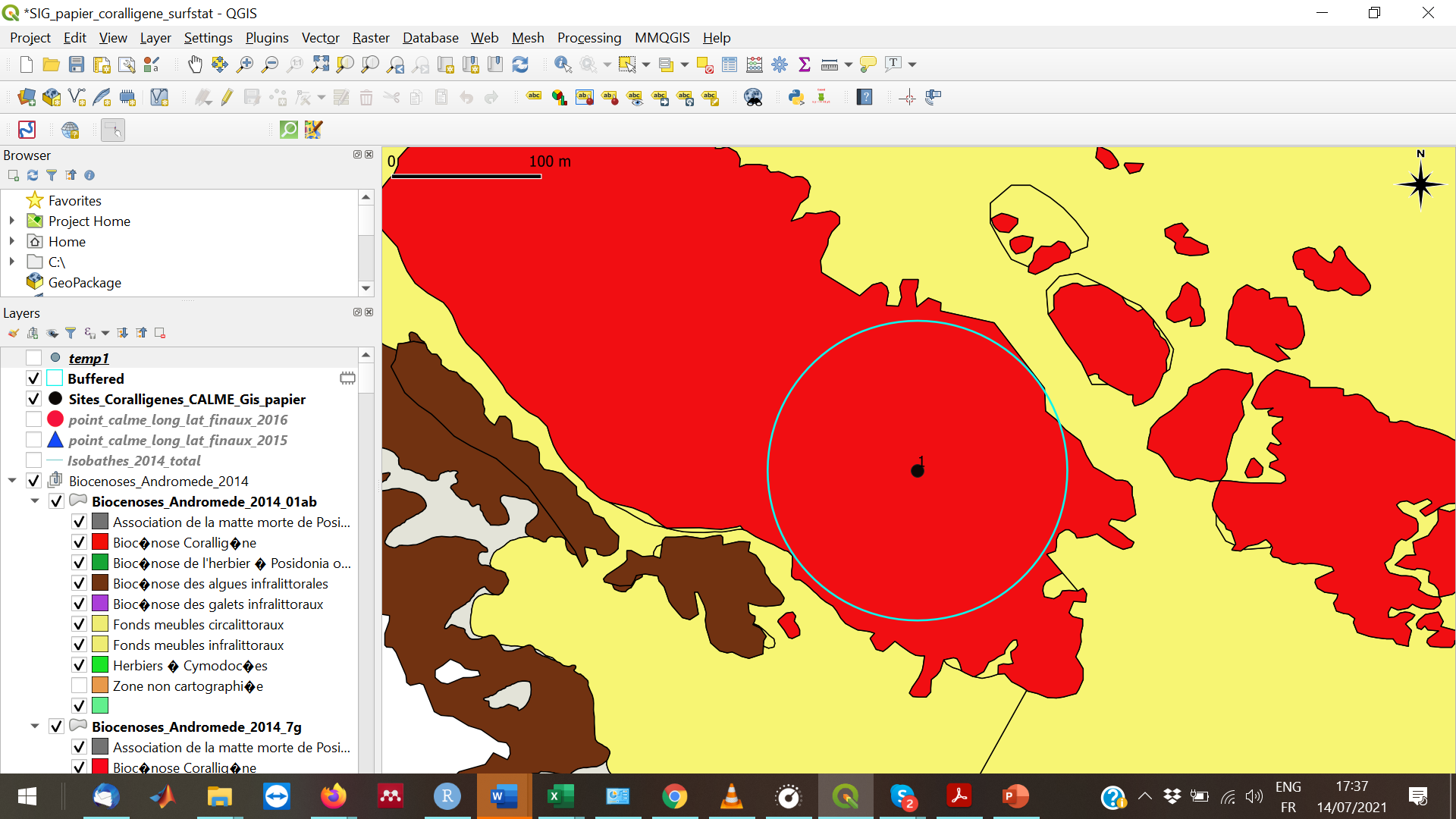

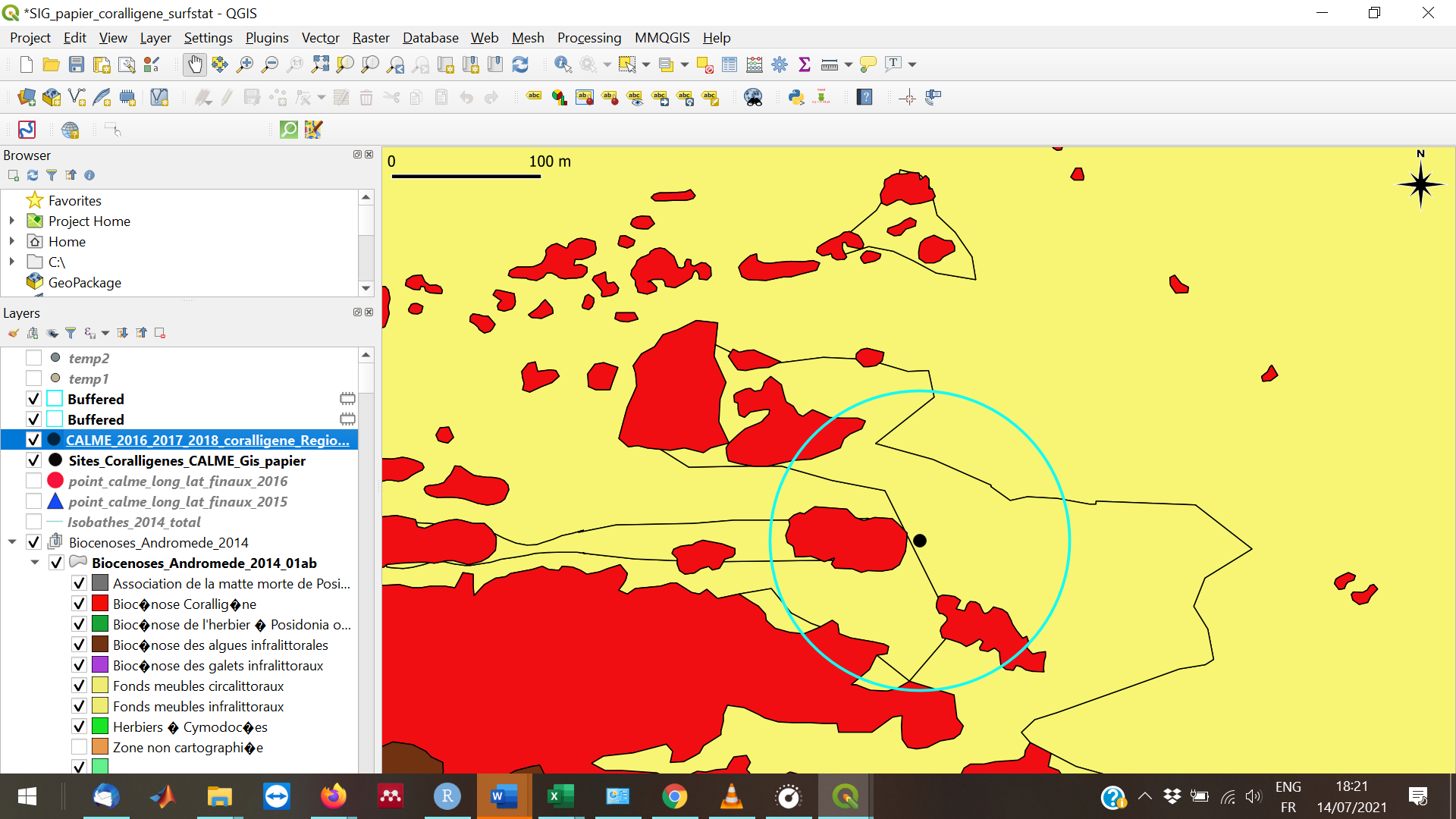


3 – Agde 4 - Grand Travers


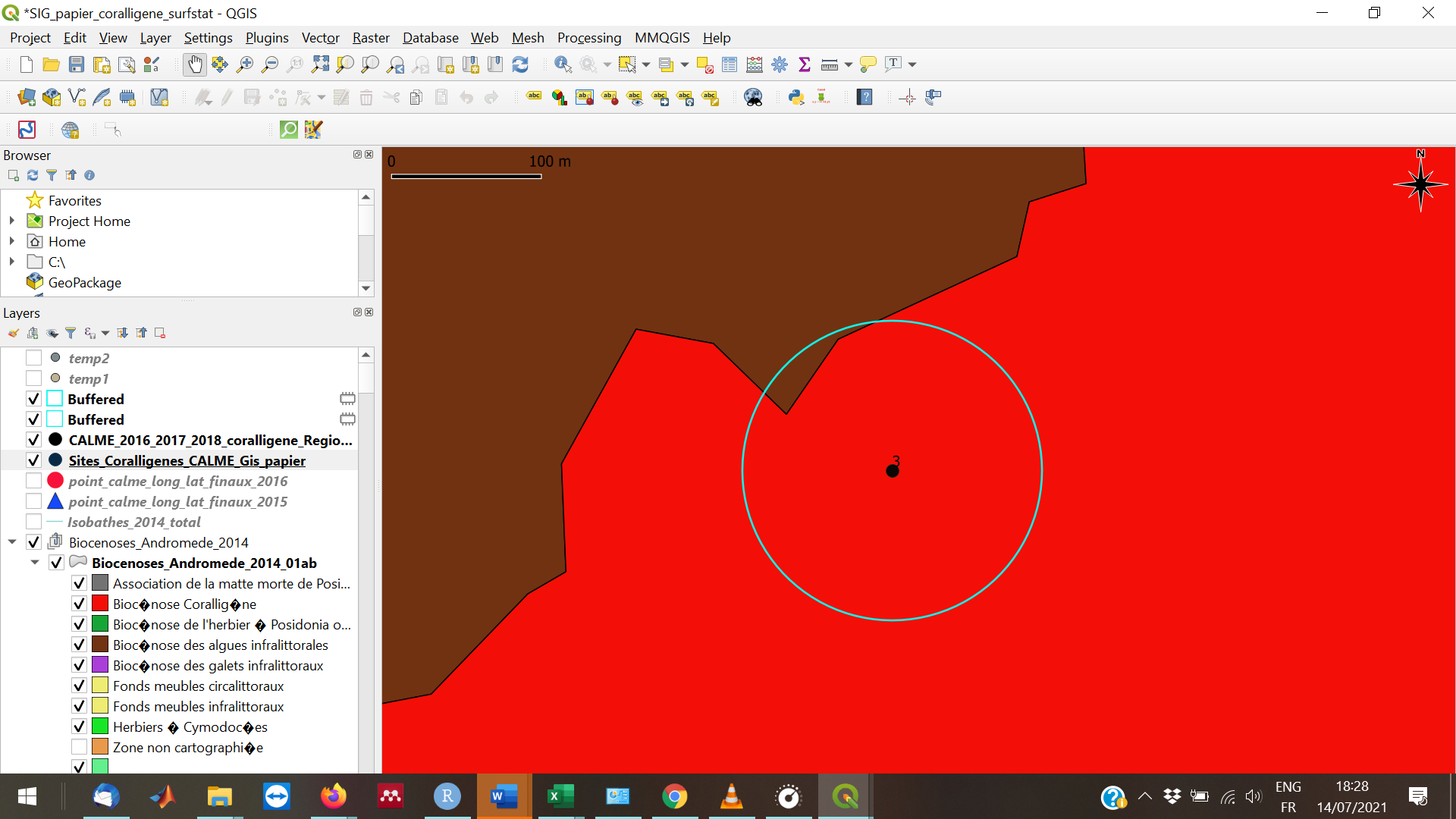

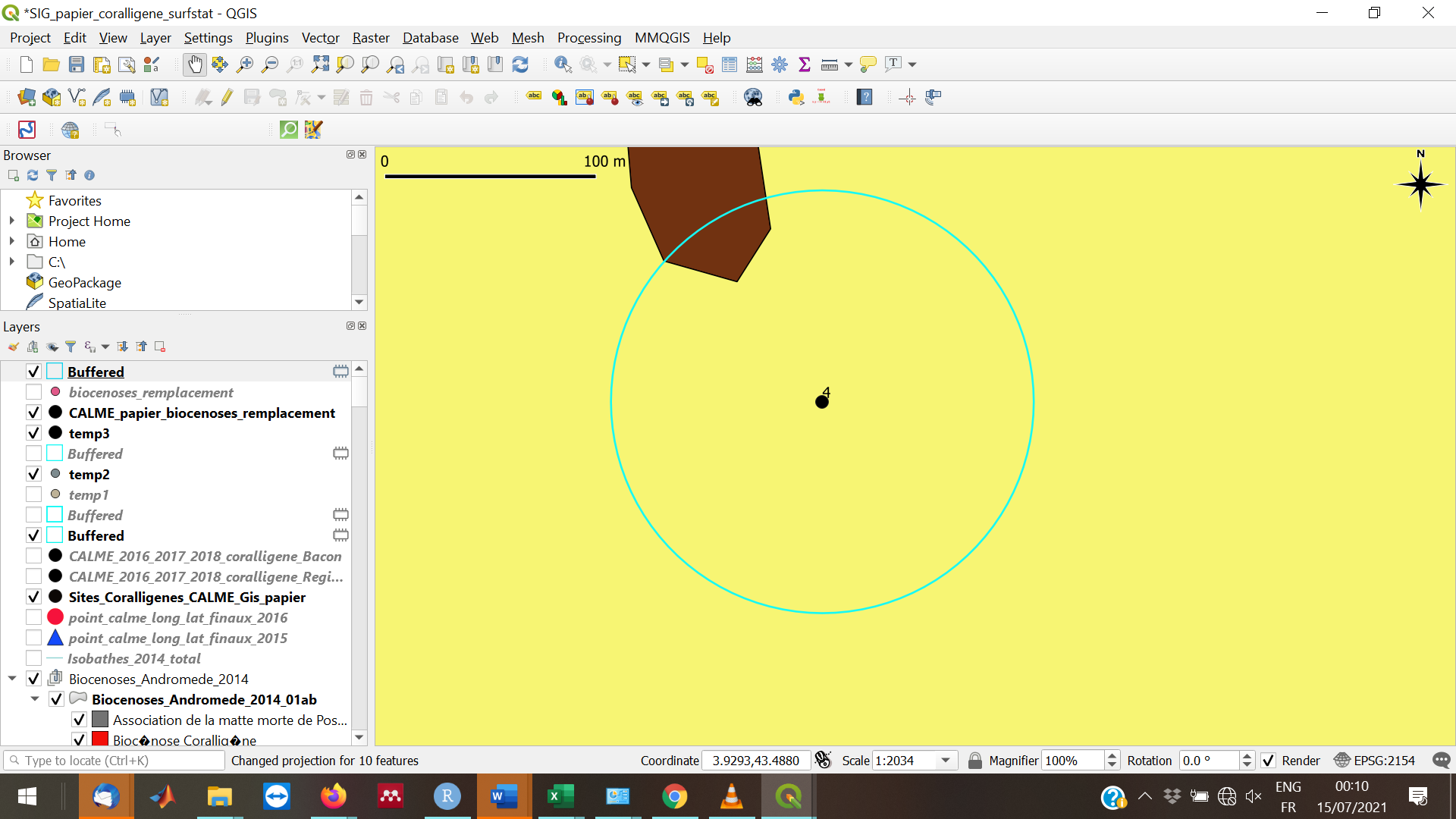


5 – Frioul 6 - Ciotat


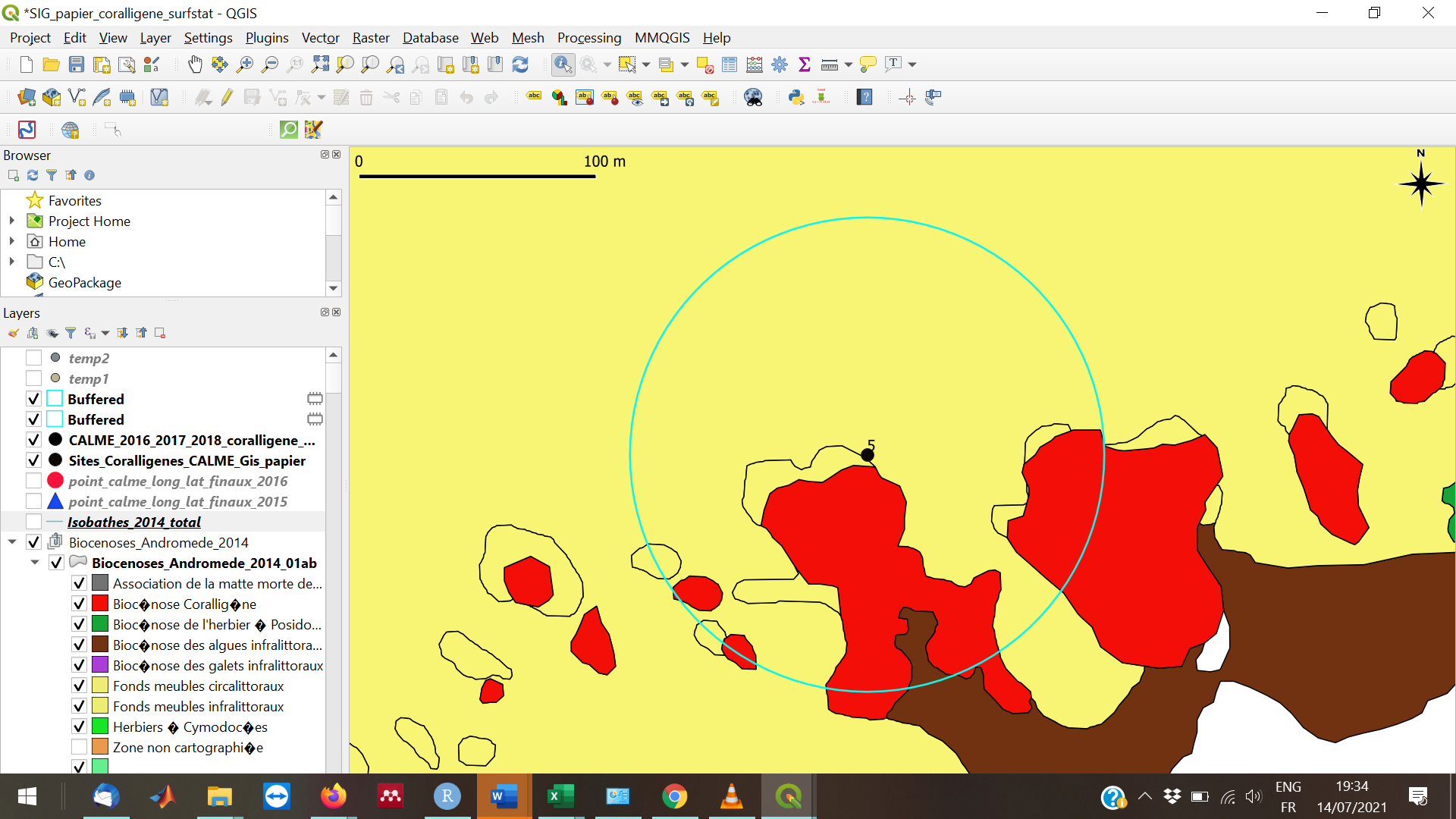

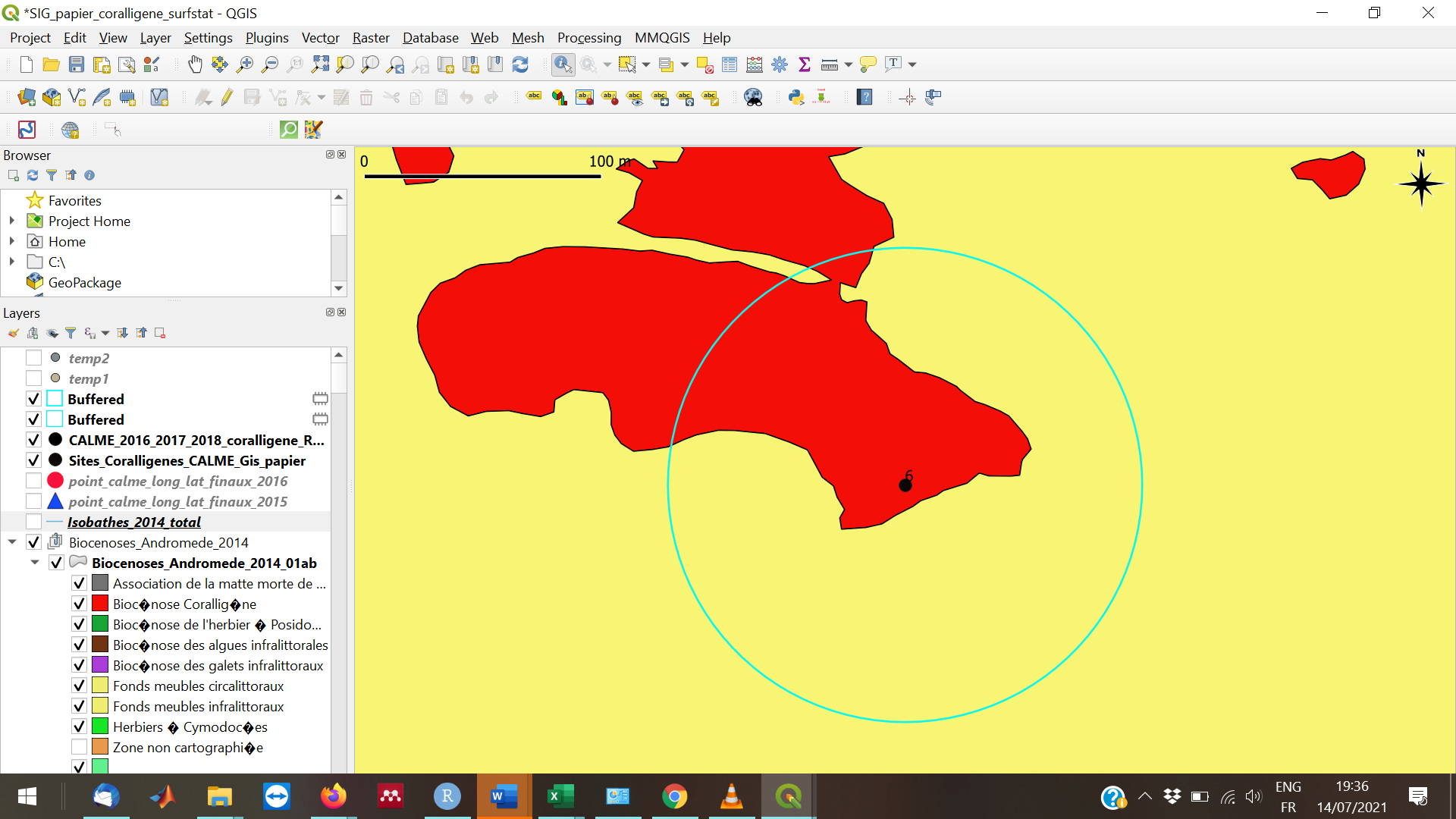


7 – Sicié 8 - Giens


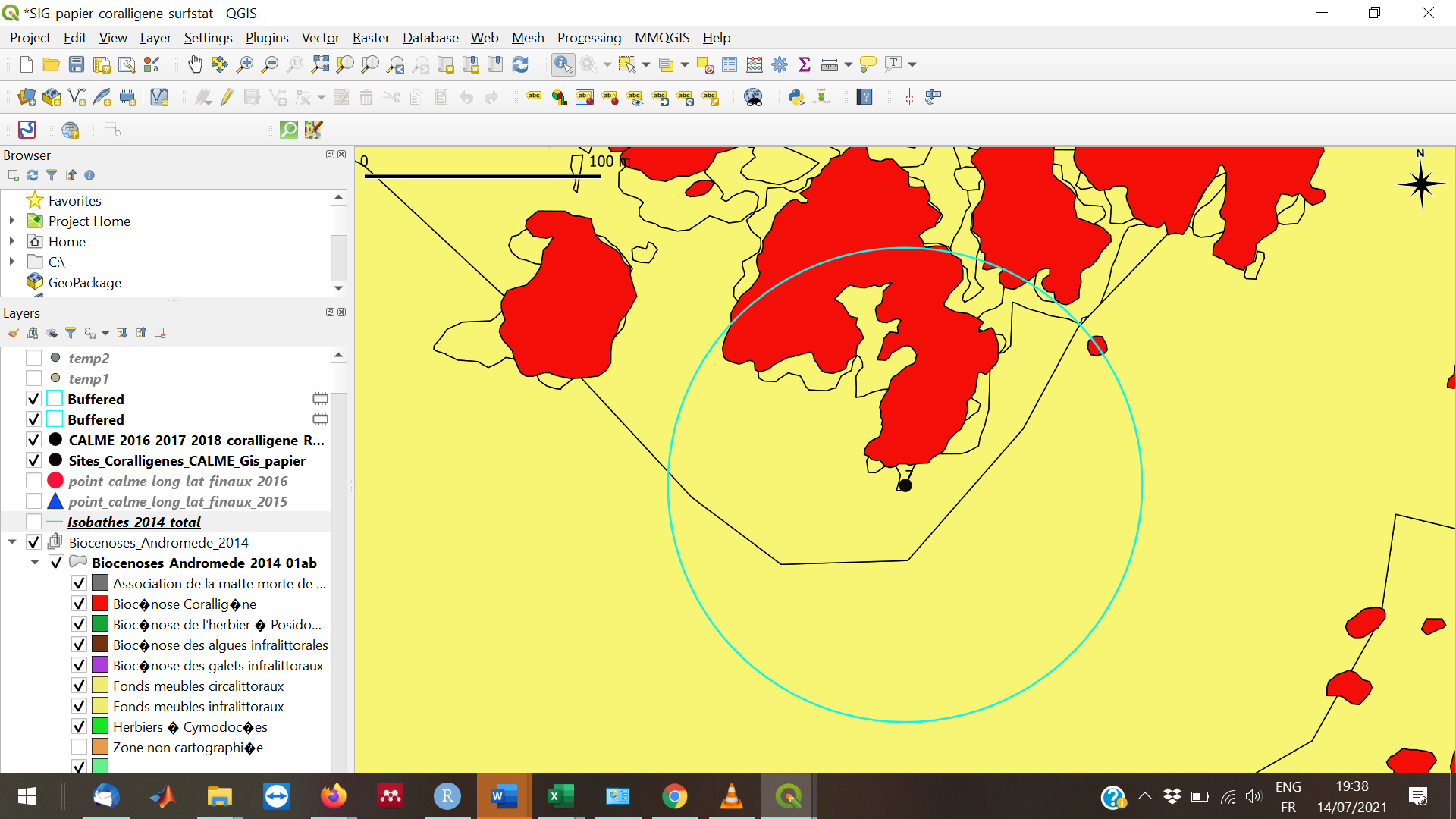

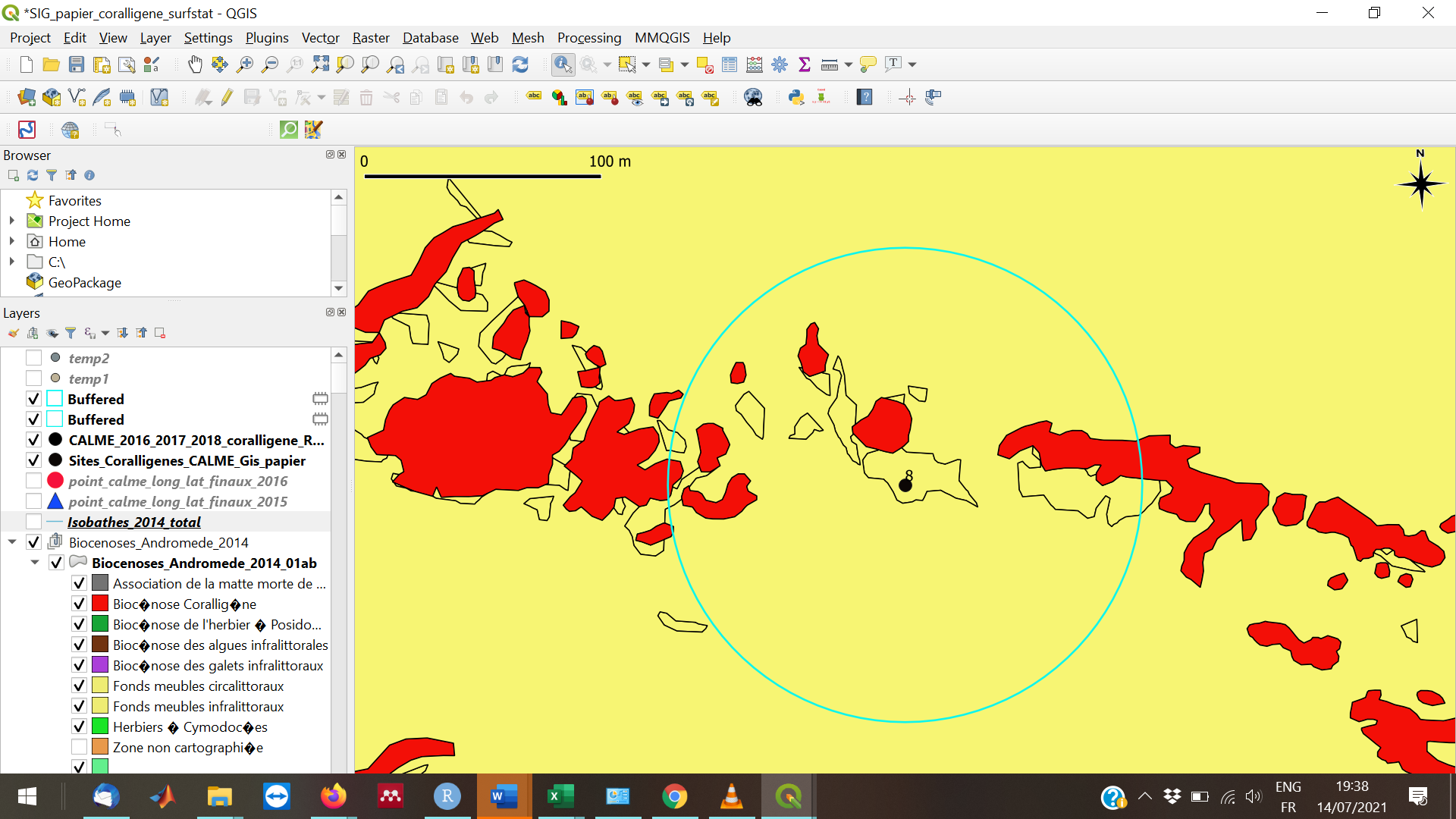


9 – Bormes 10 - Cap Lardier


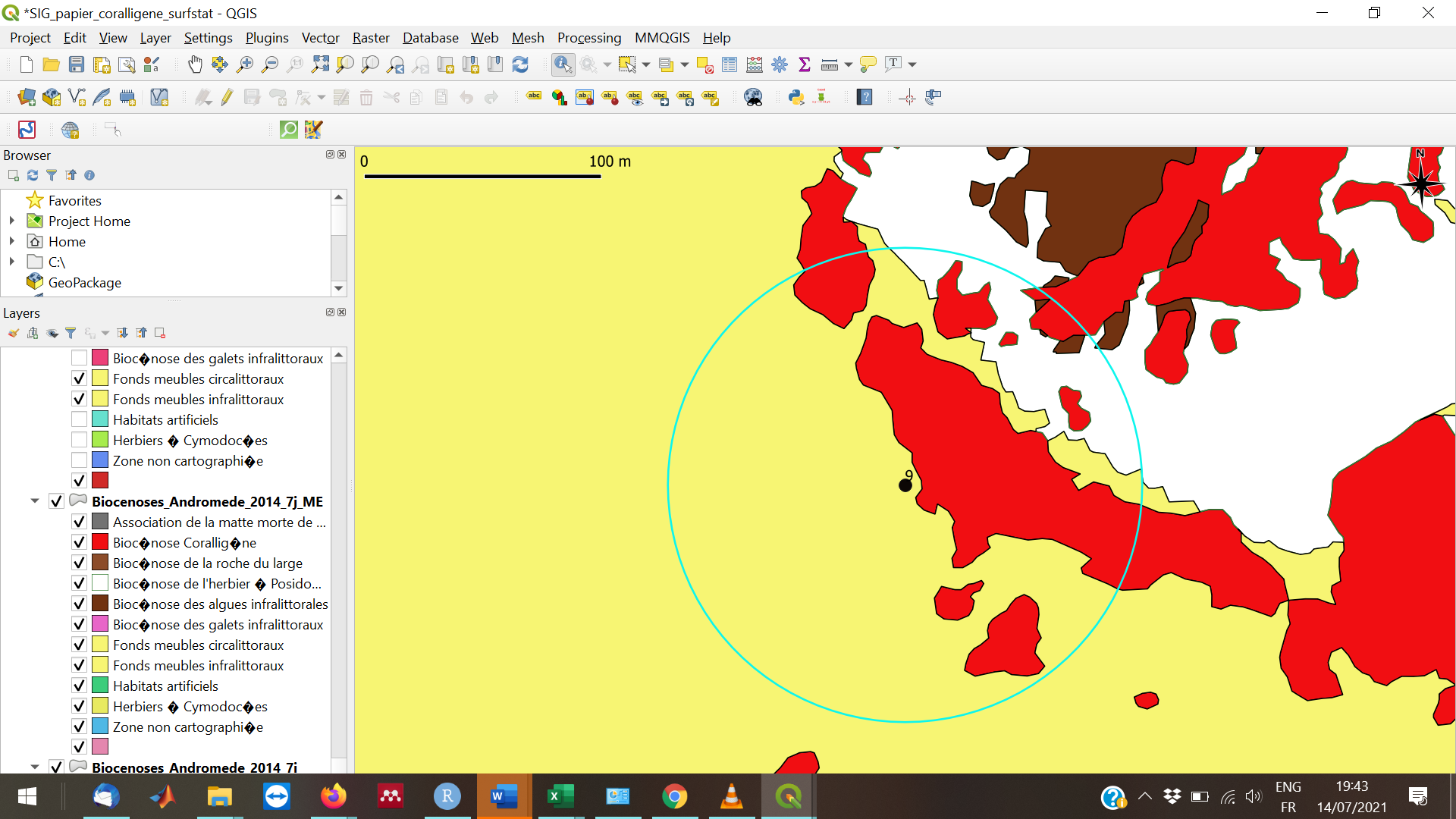

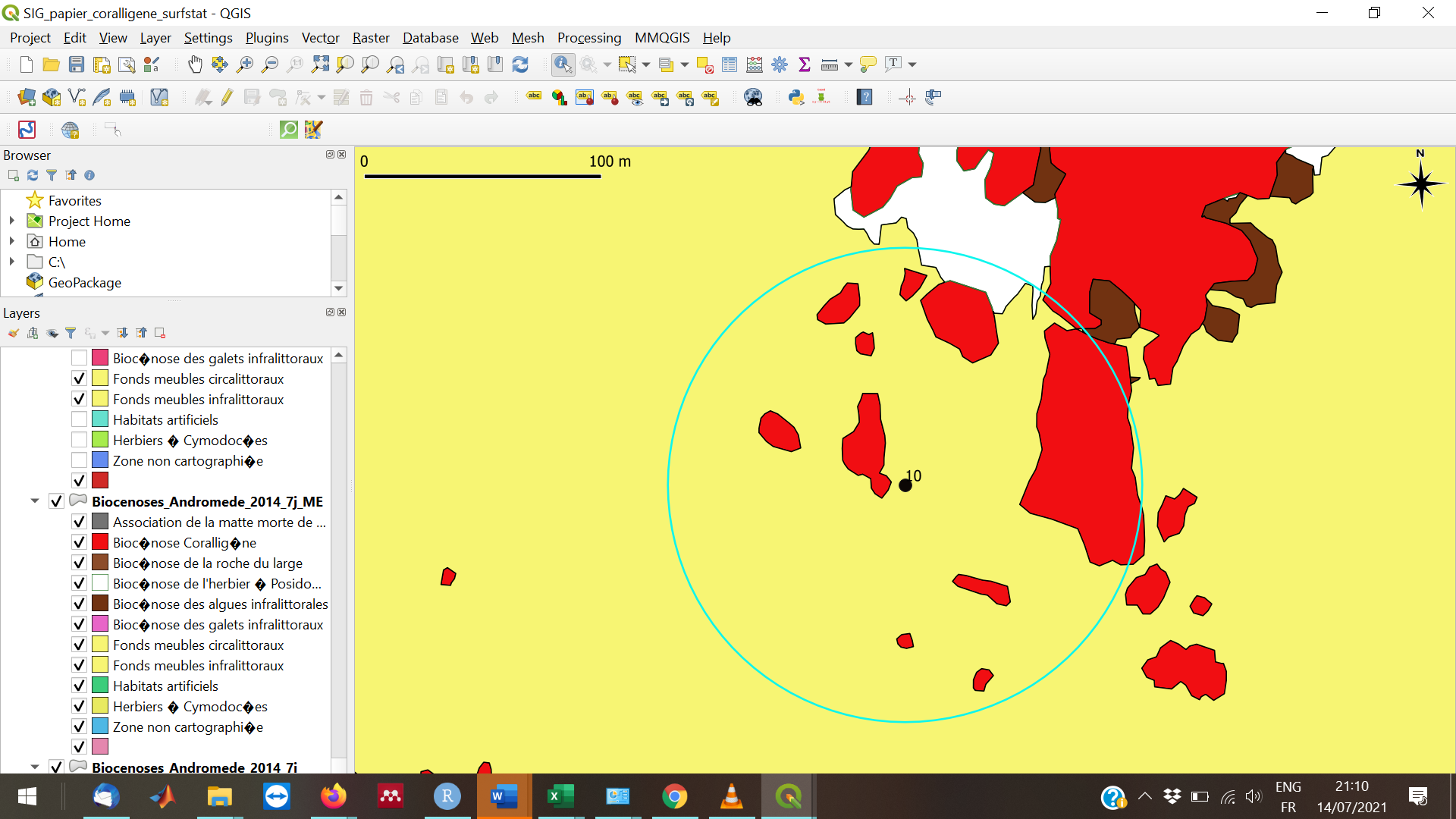


11 – Bonneau 12 - Cap Roux


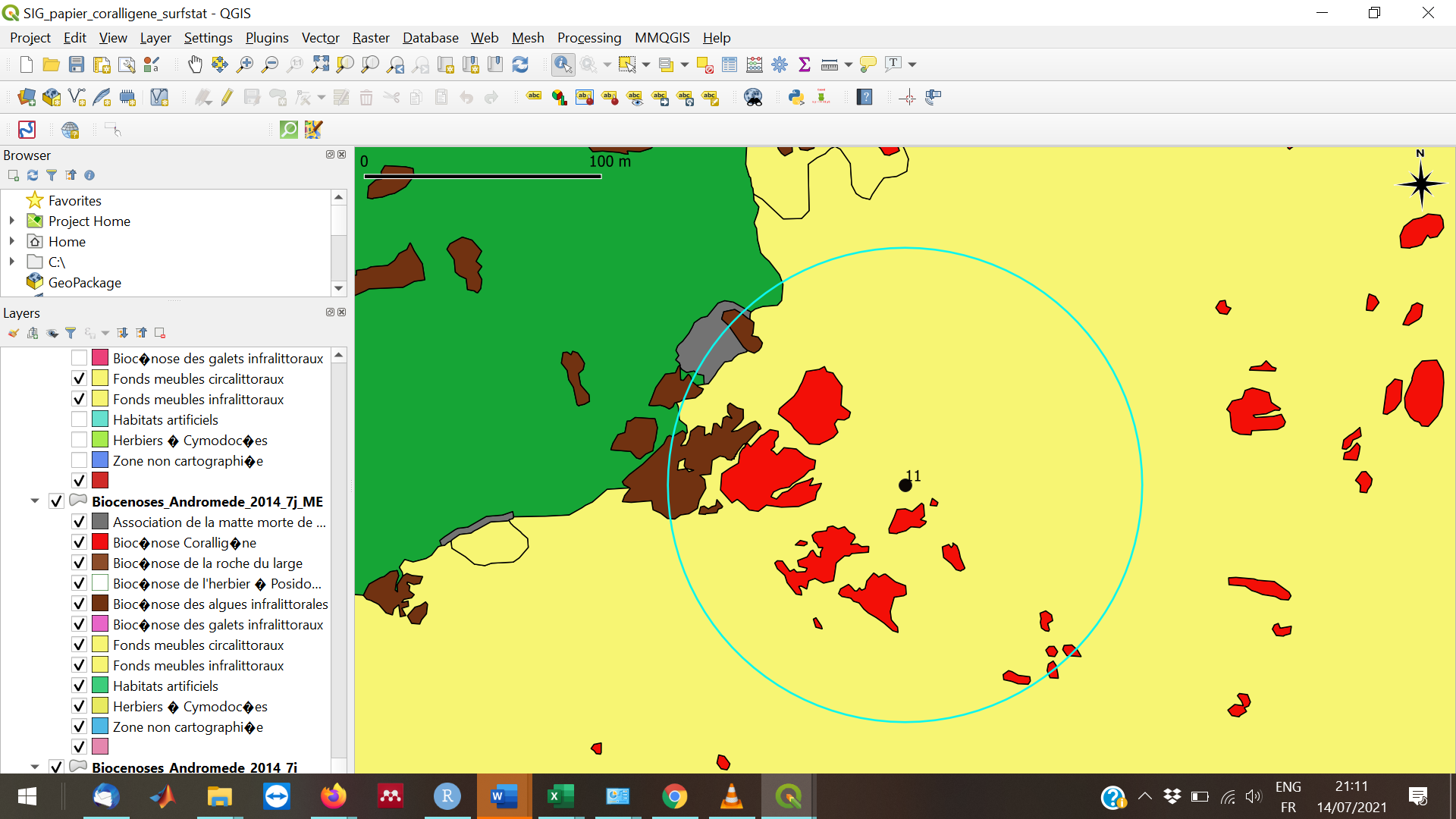

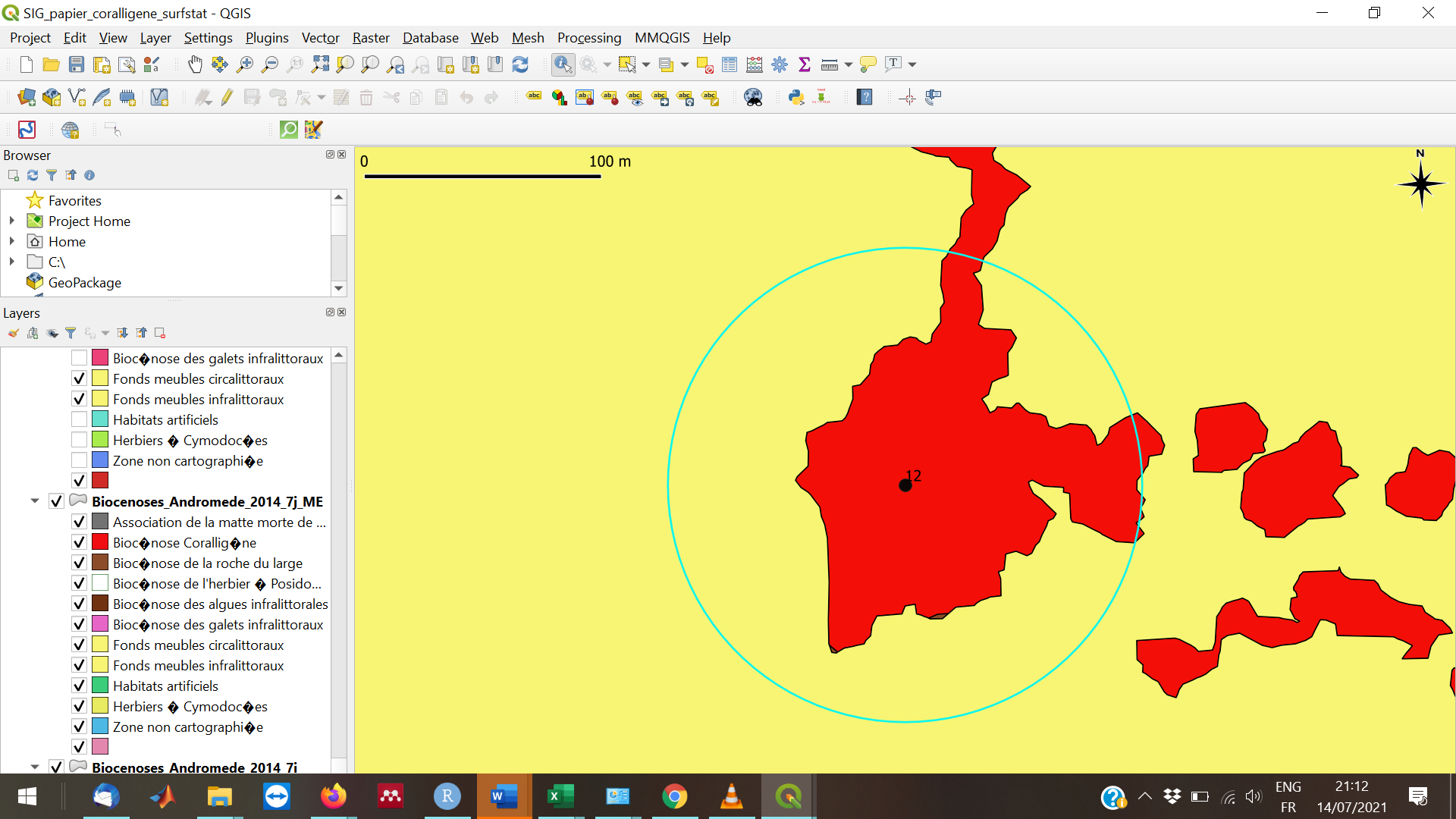


13 – Golfe Juan 14 - Bacon


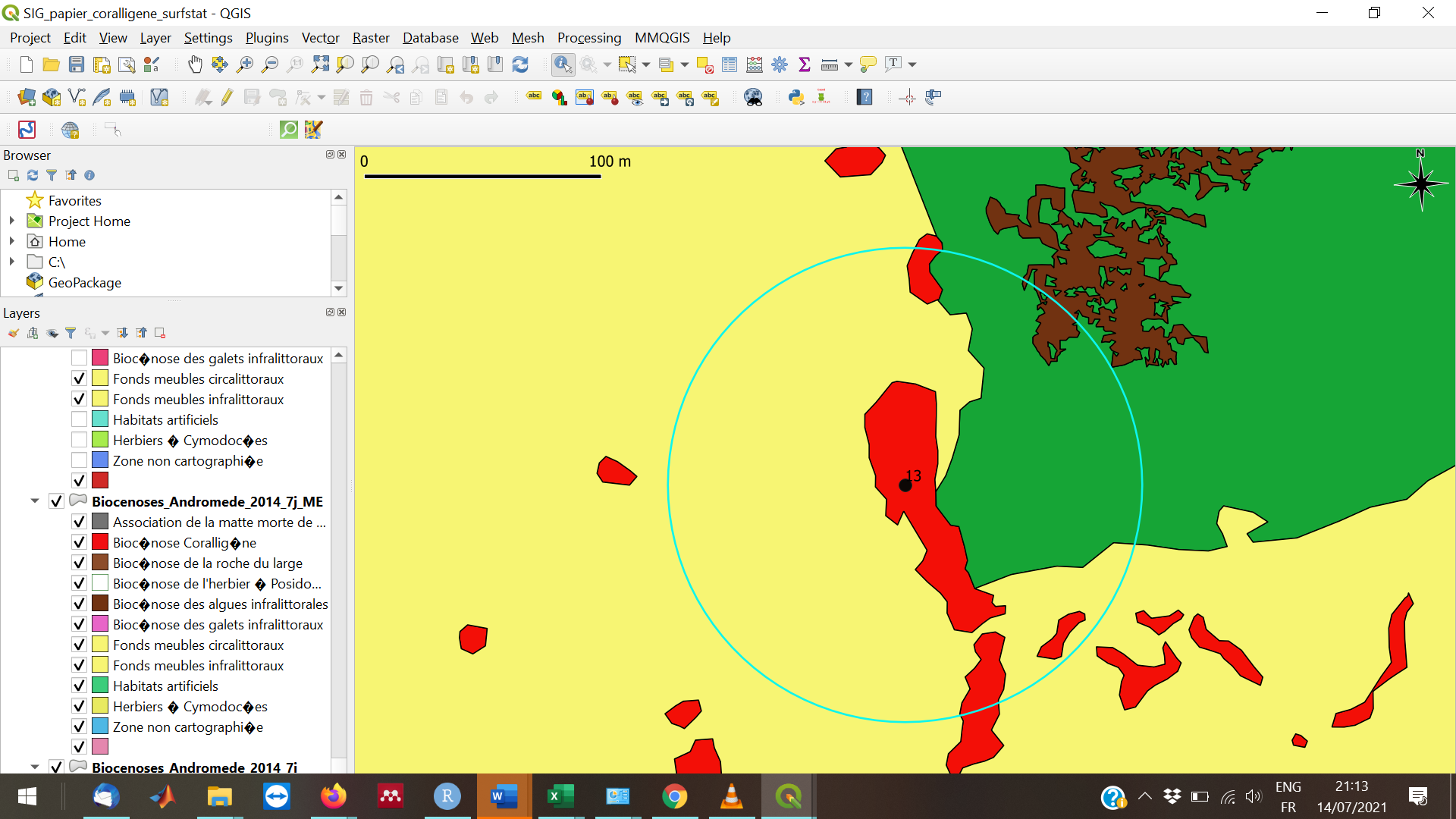

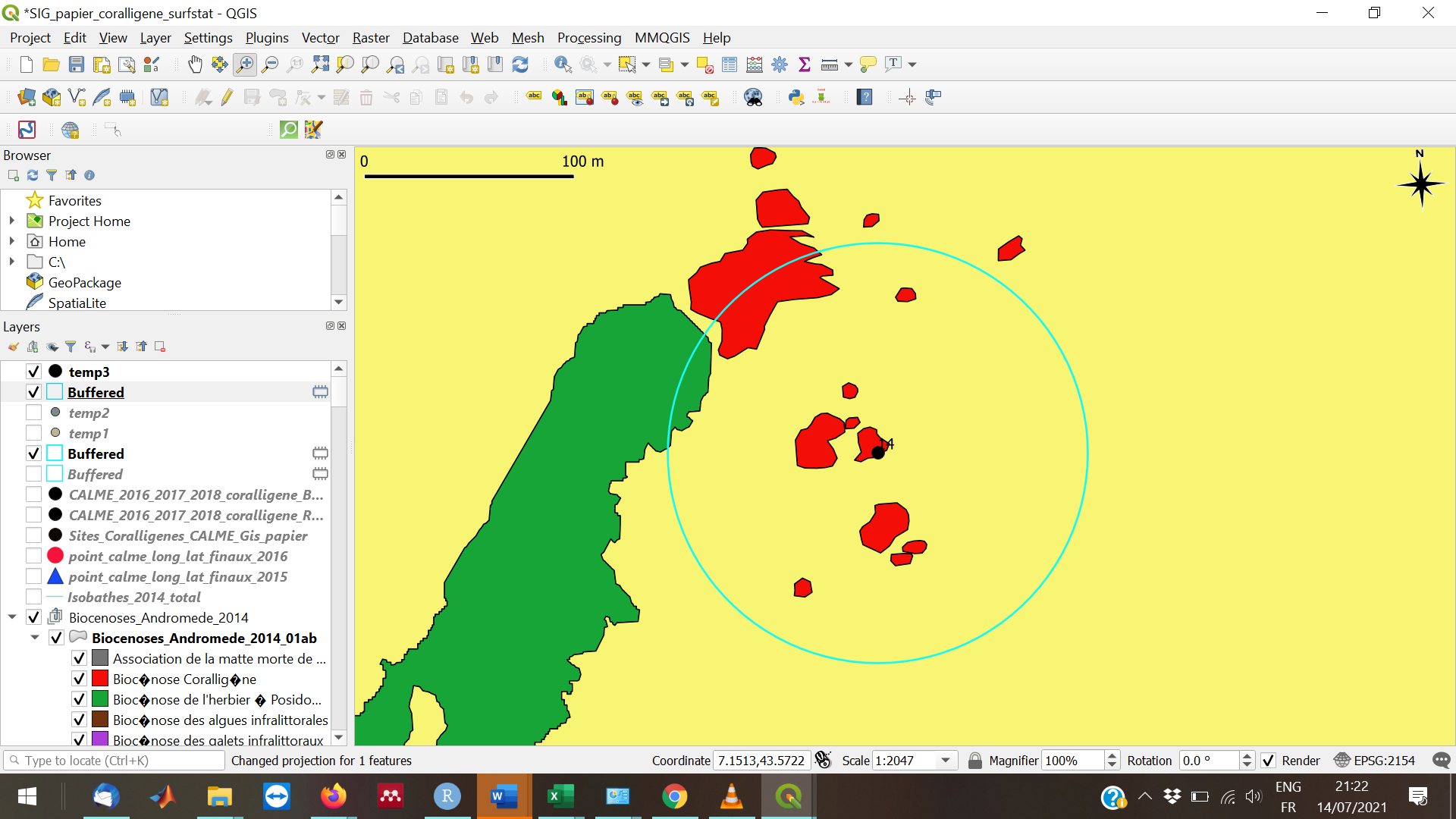


15 – Tombants Américains 16 - Eze


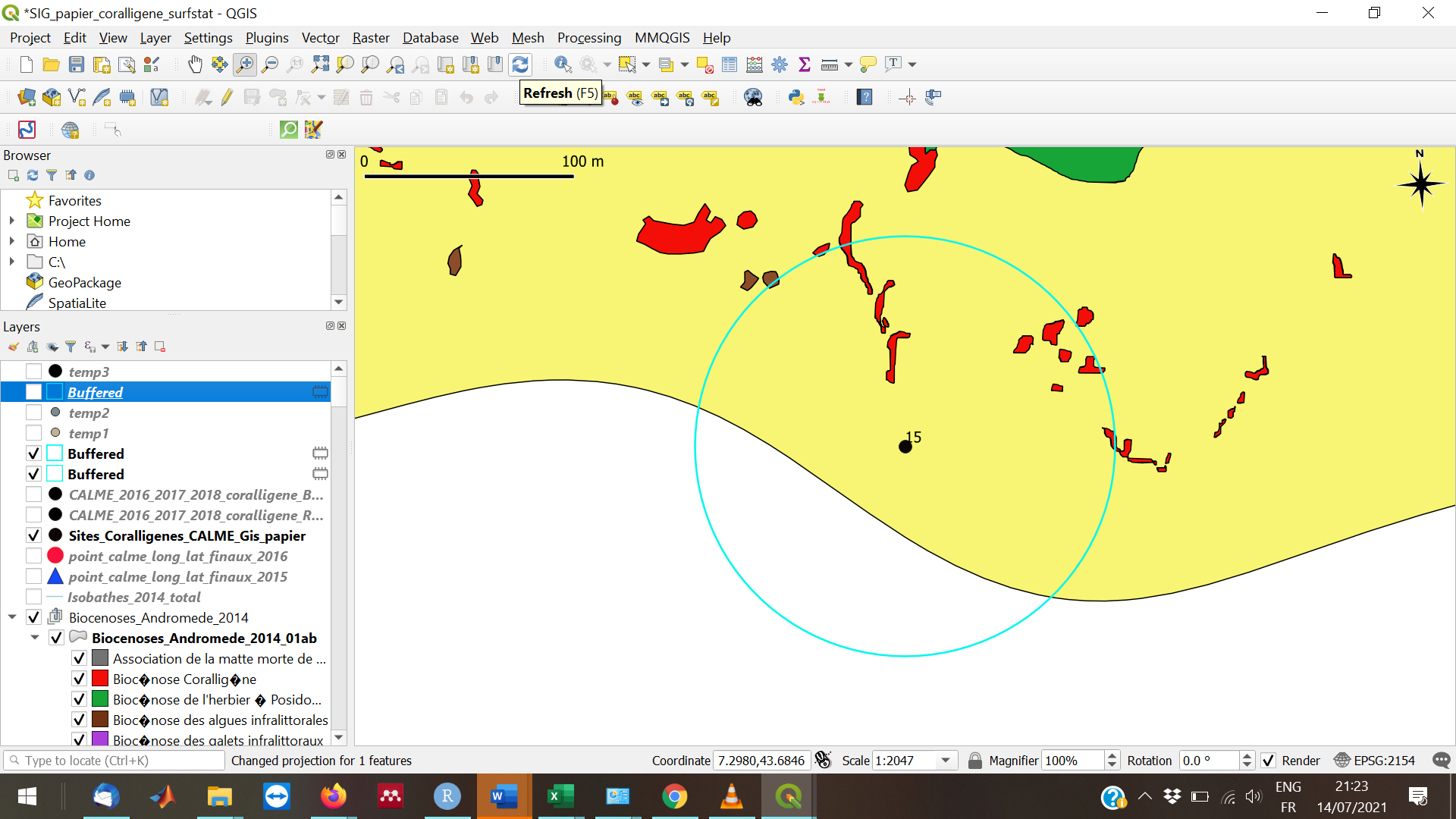

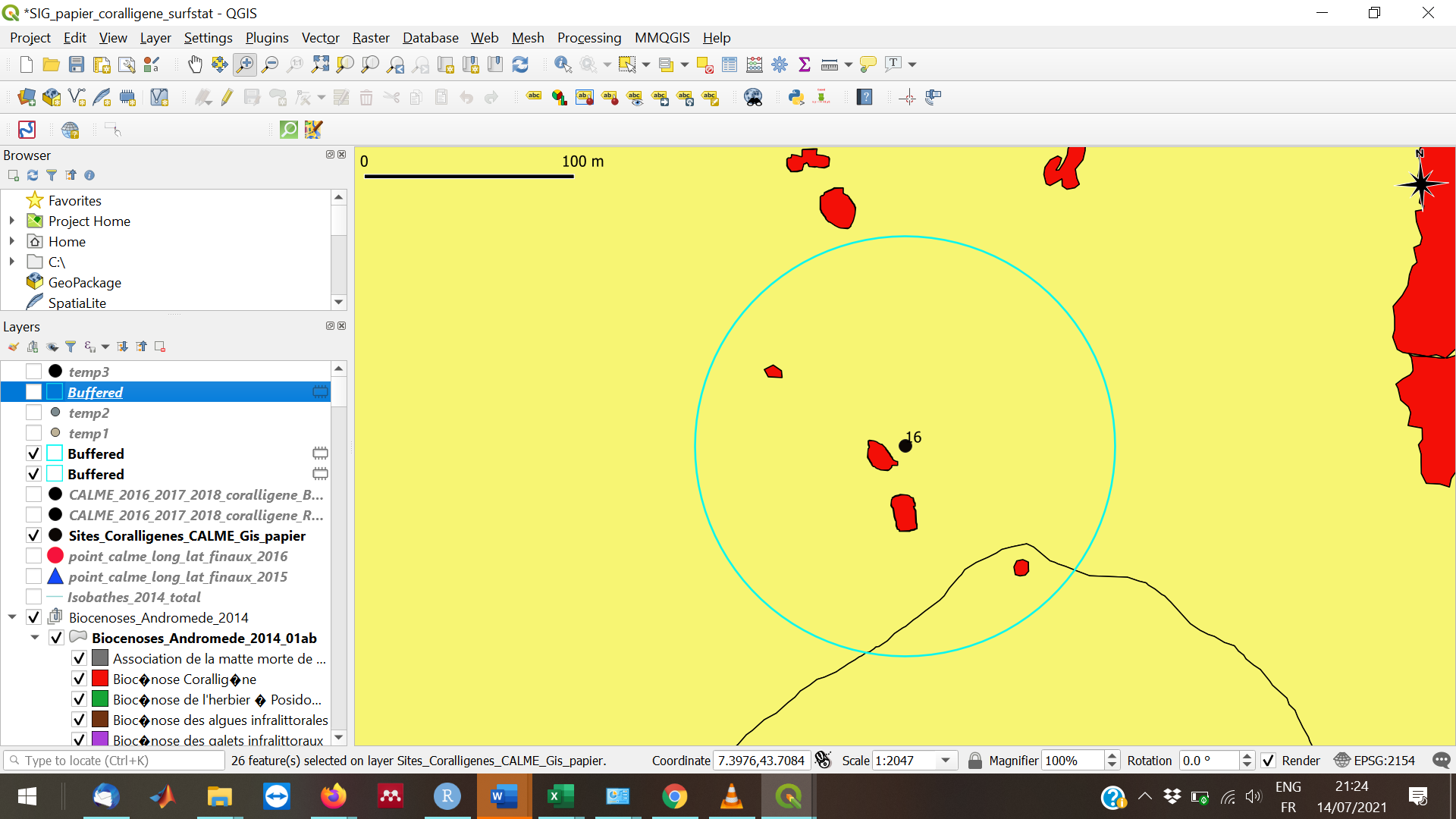


17 – Cap Martin 18 - Maccinagio


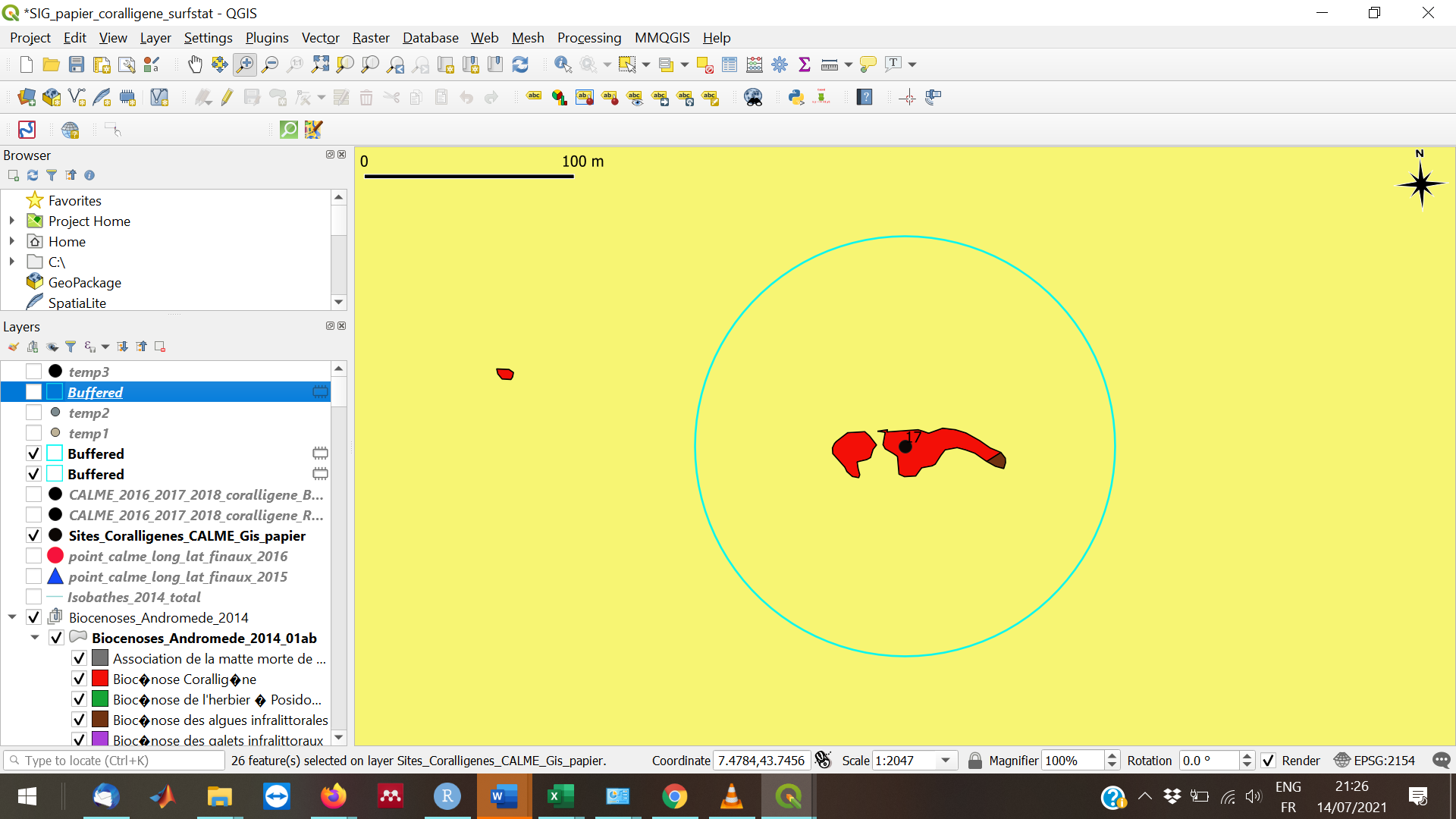

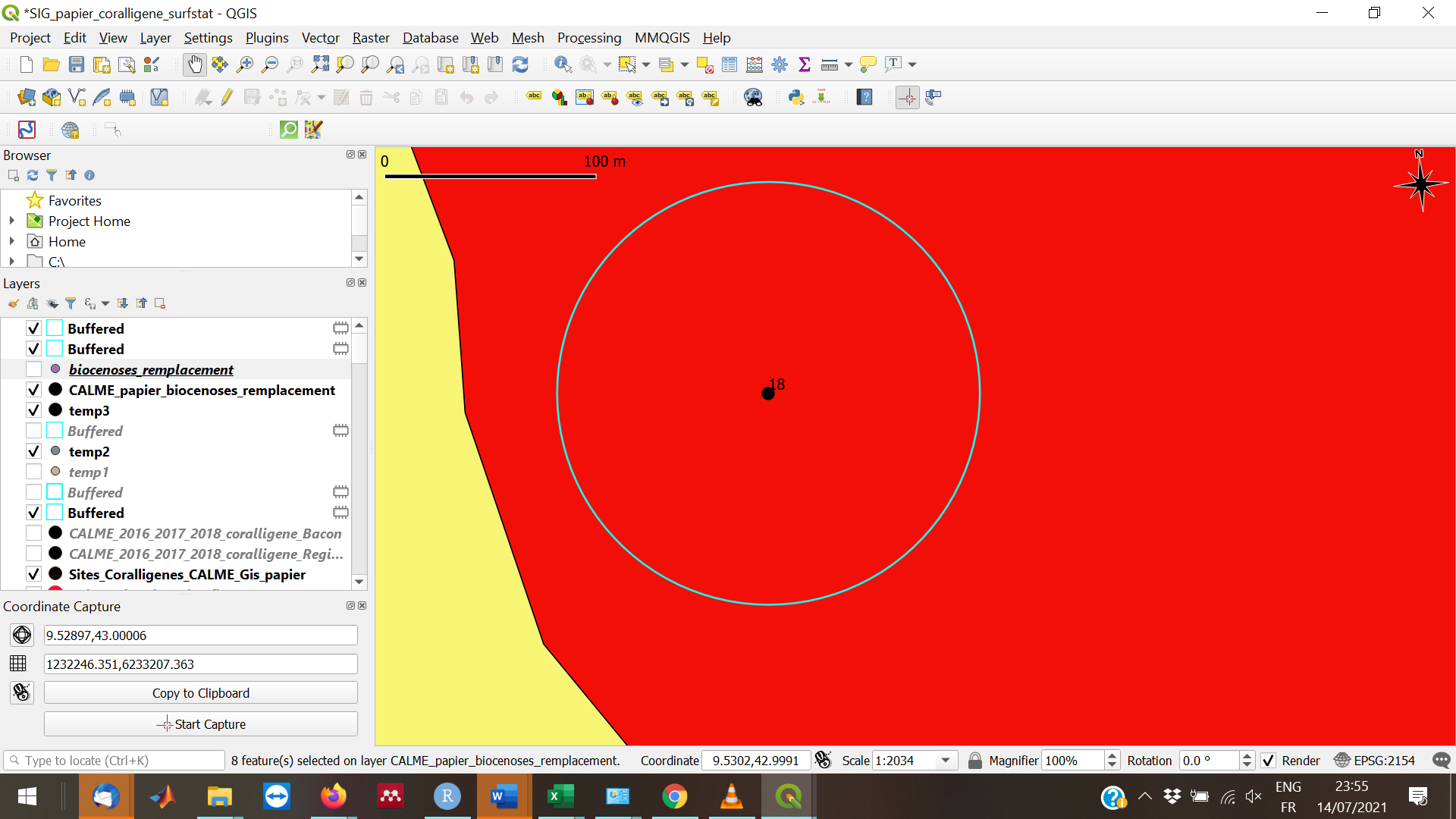


19 – Bastia 20 - Tarco


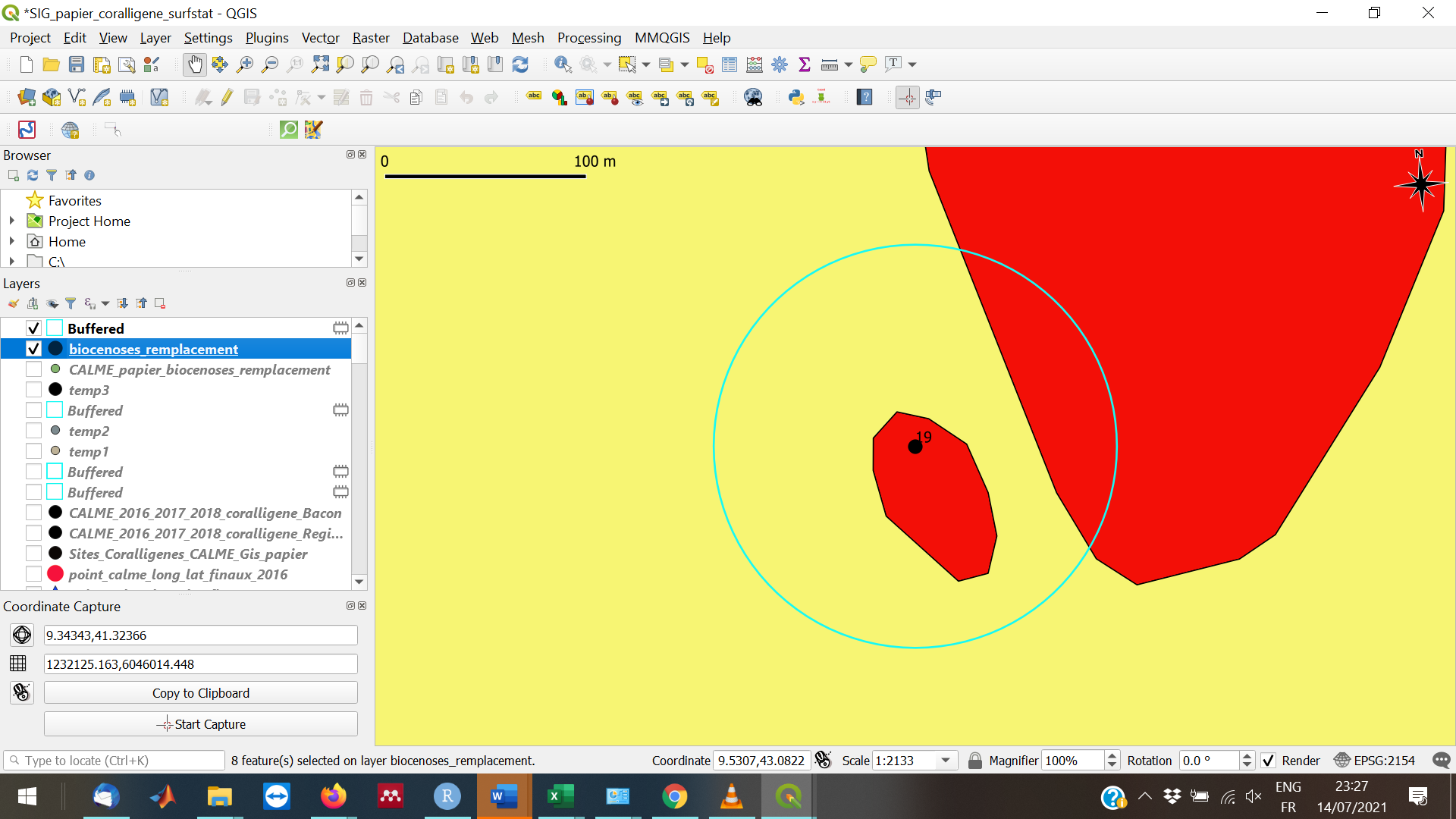

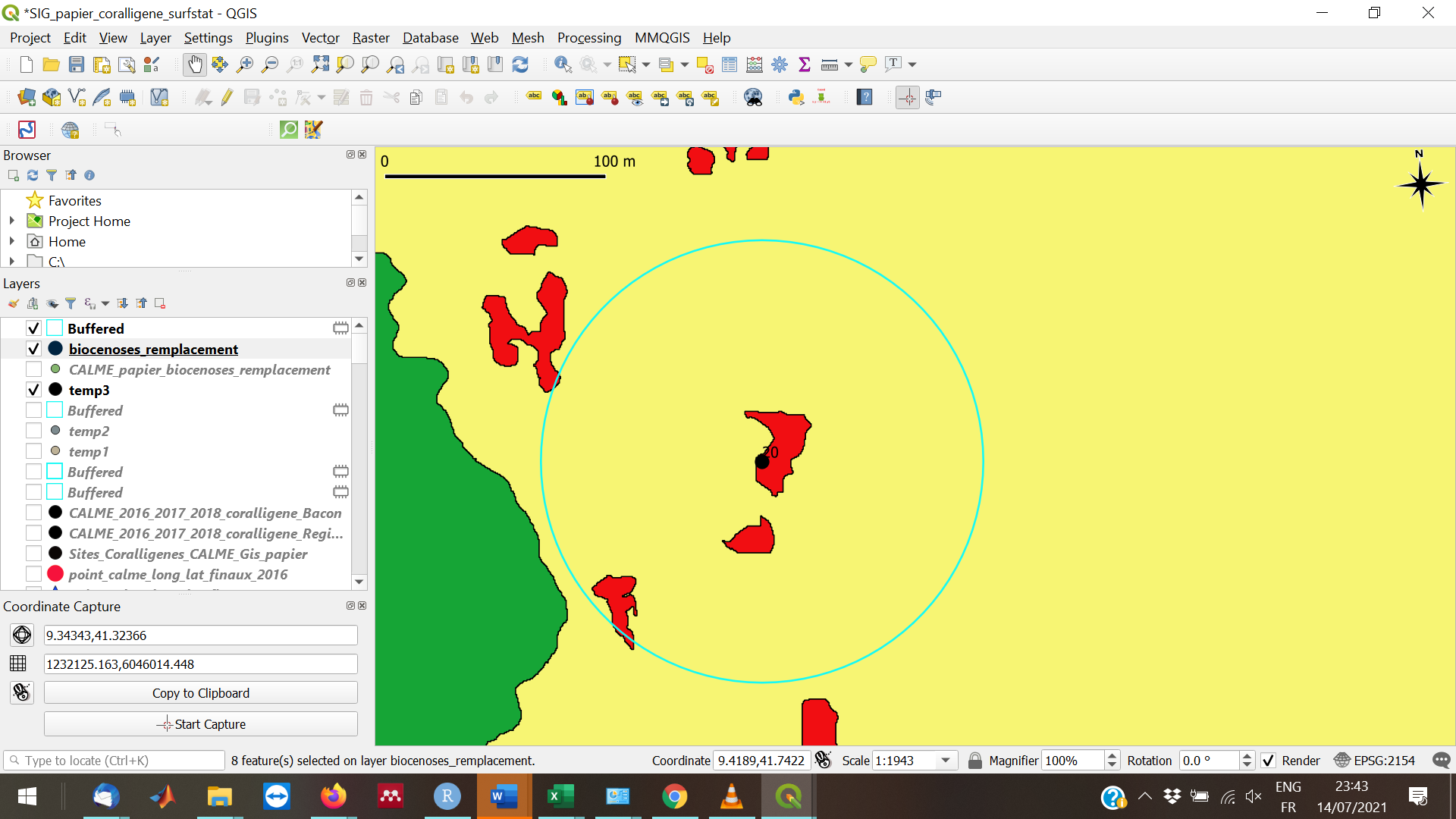


21 – Rondinara 22 - Murtoli


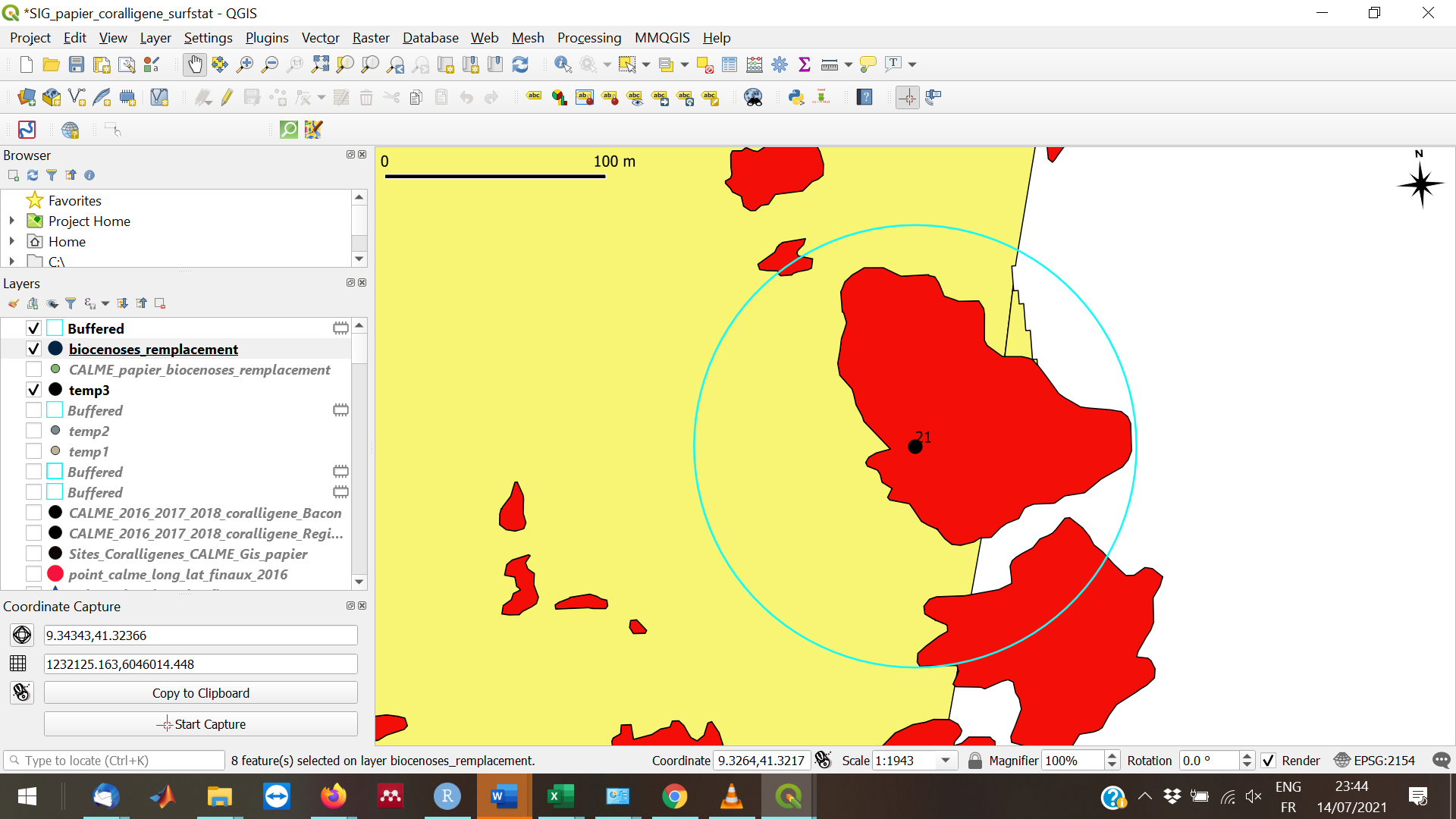

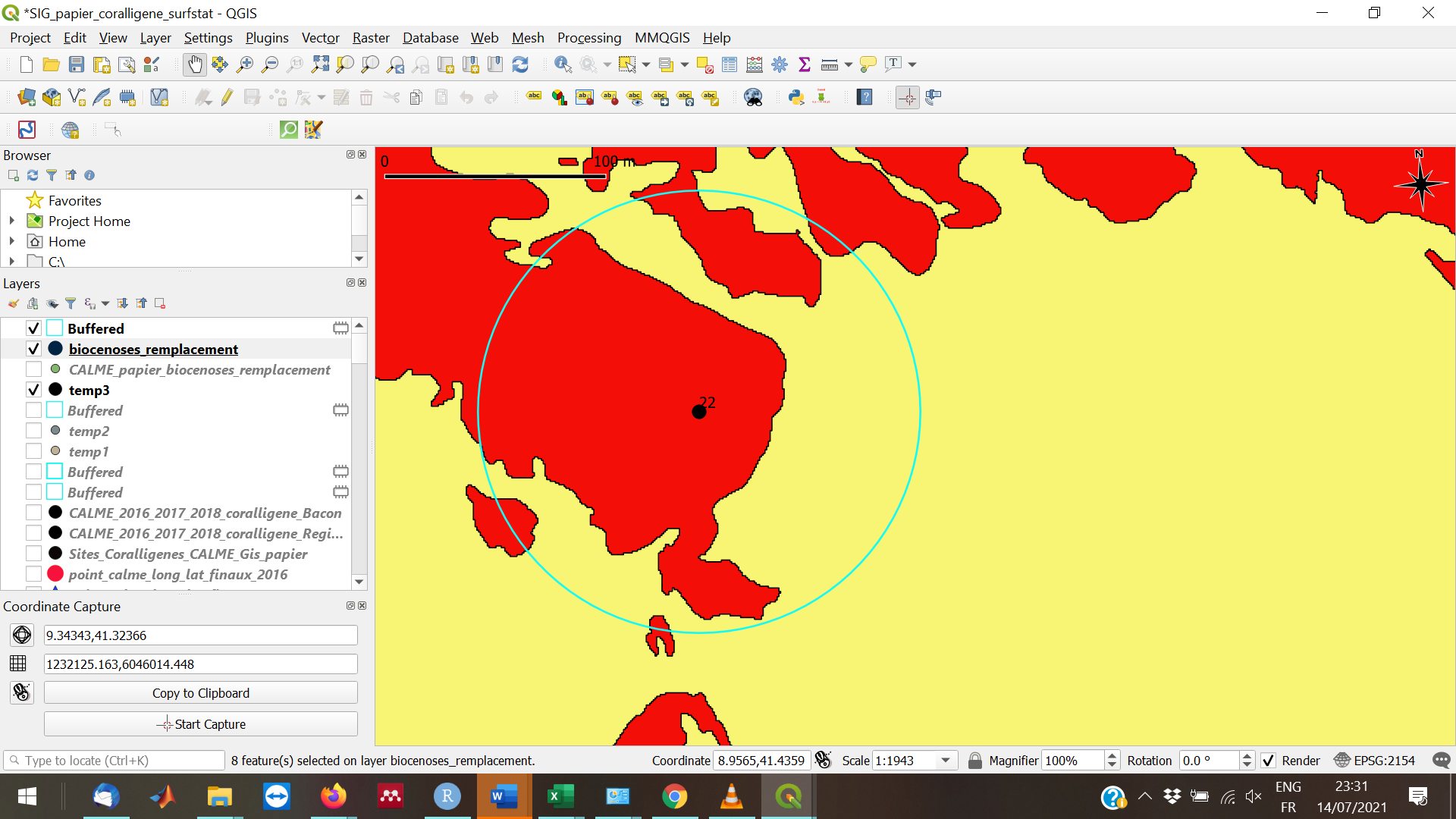


23 – Parata 24 - Cappu Rosso


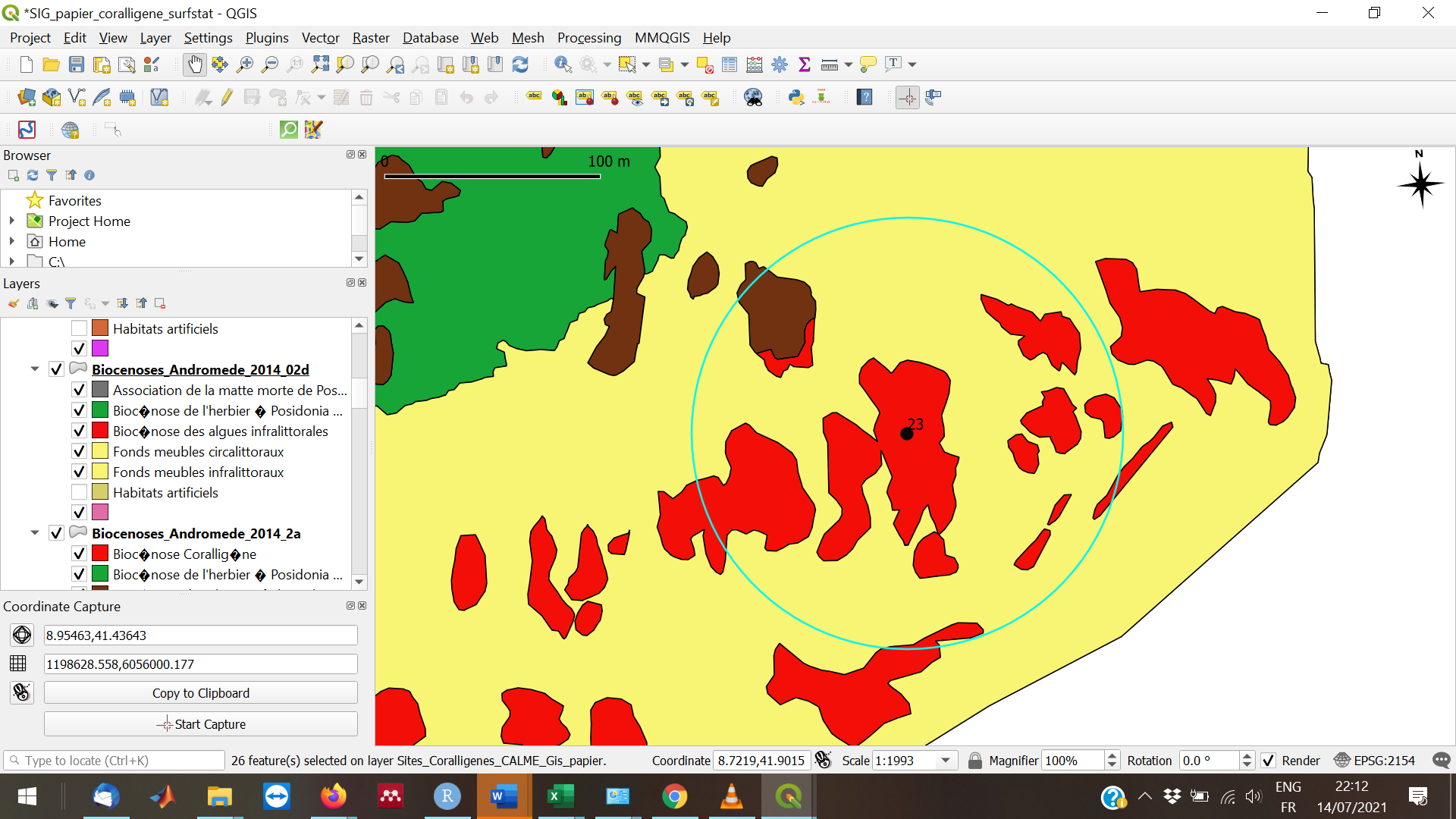

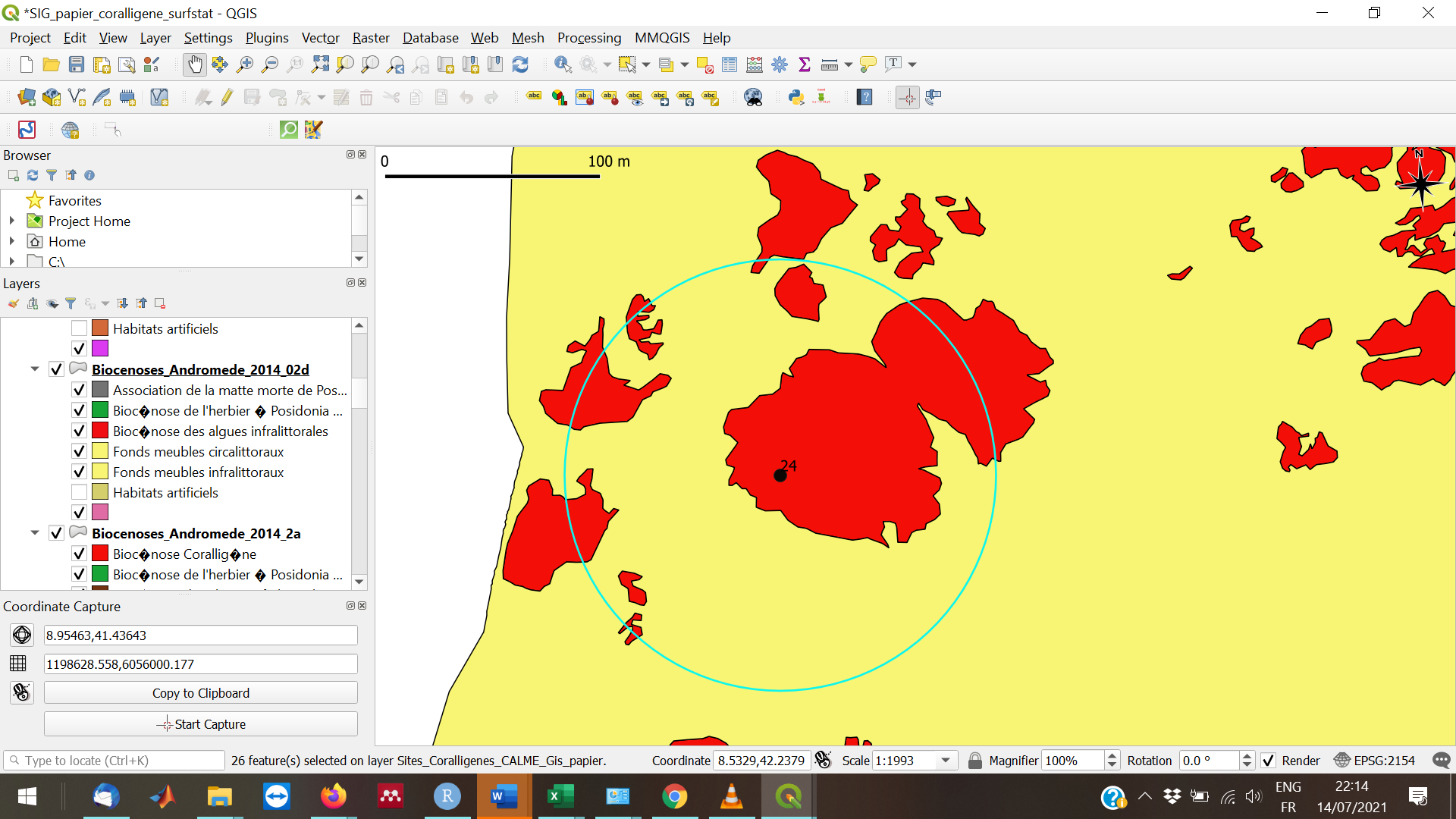


25 – Focolara 26 - Calvi


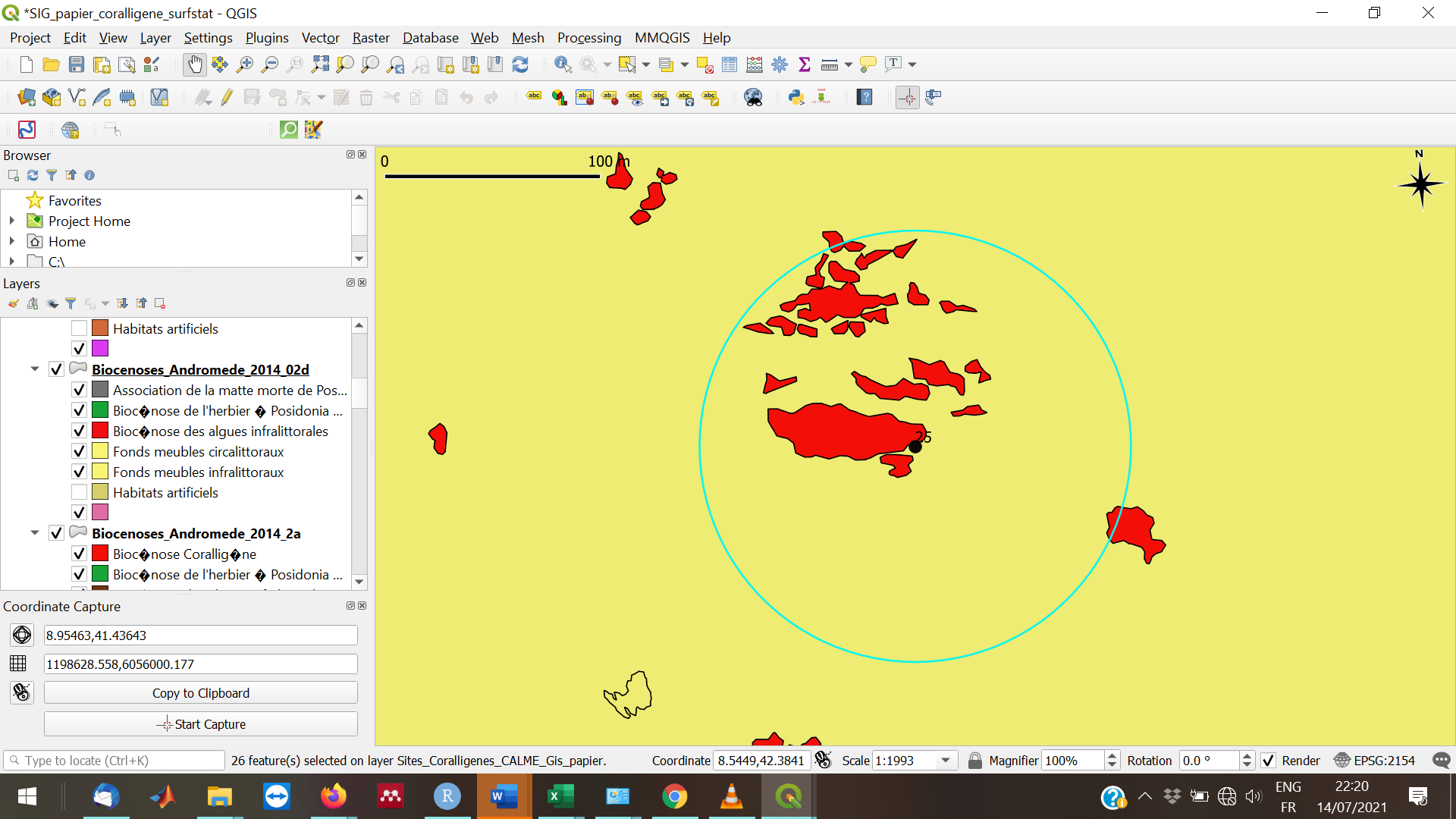

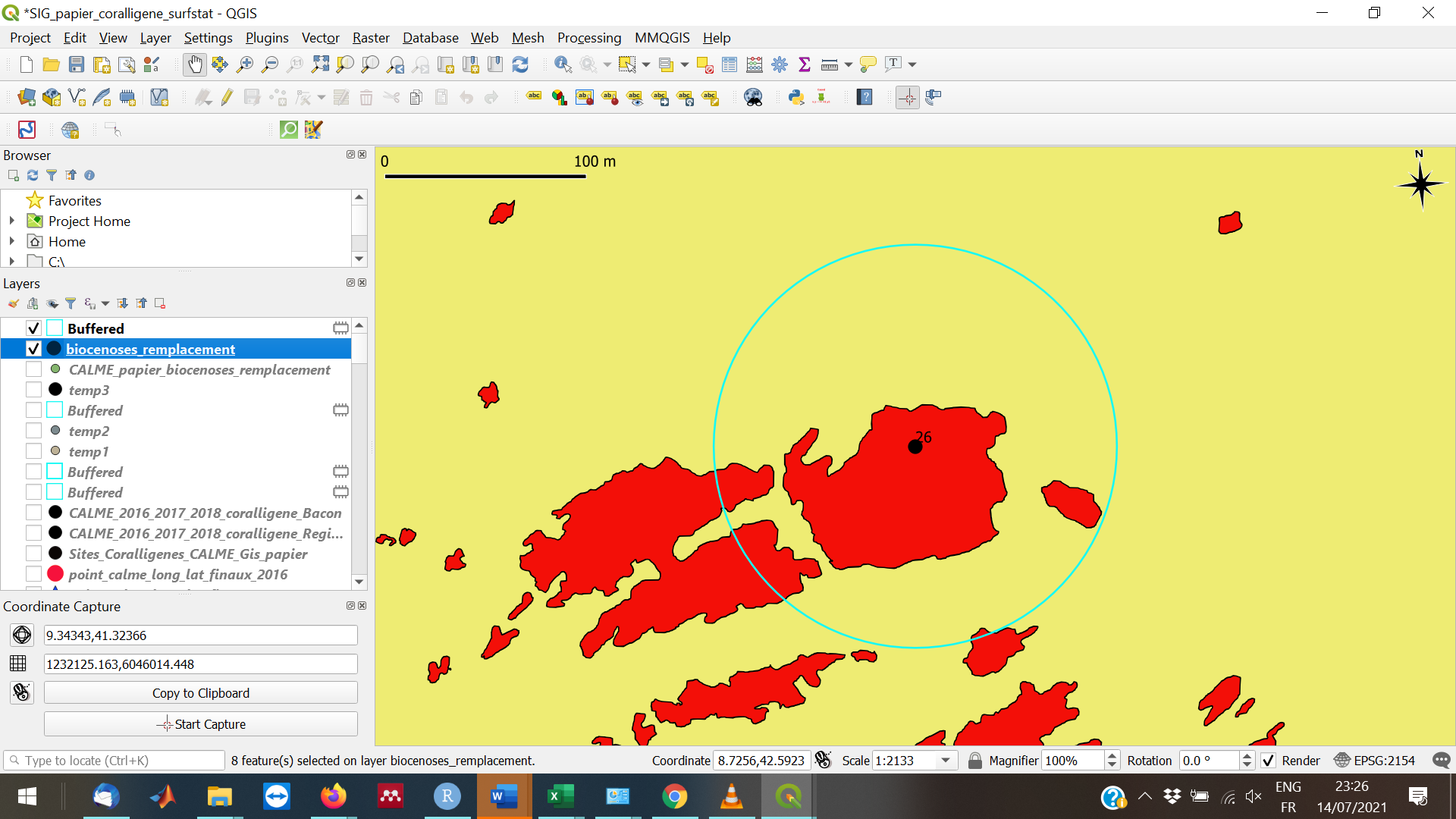


27 – Agriates


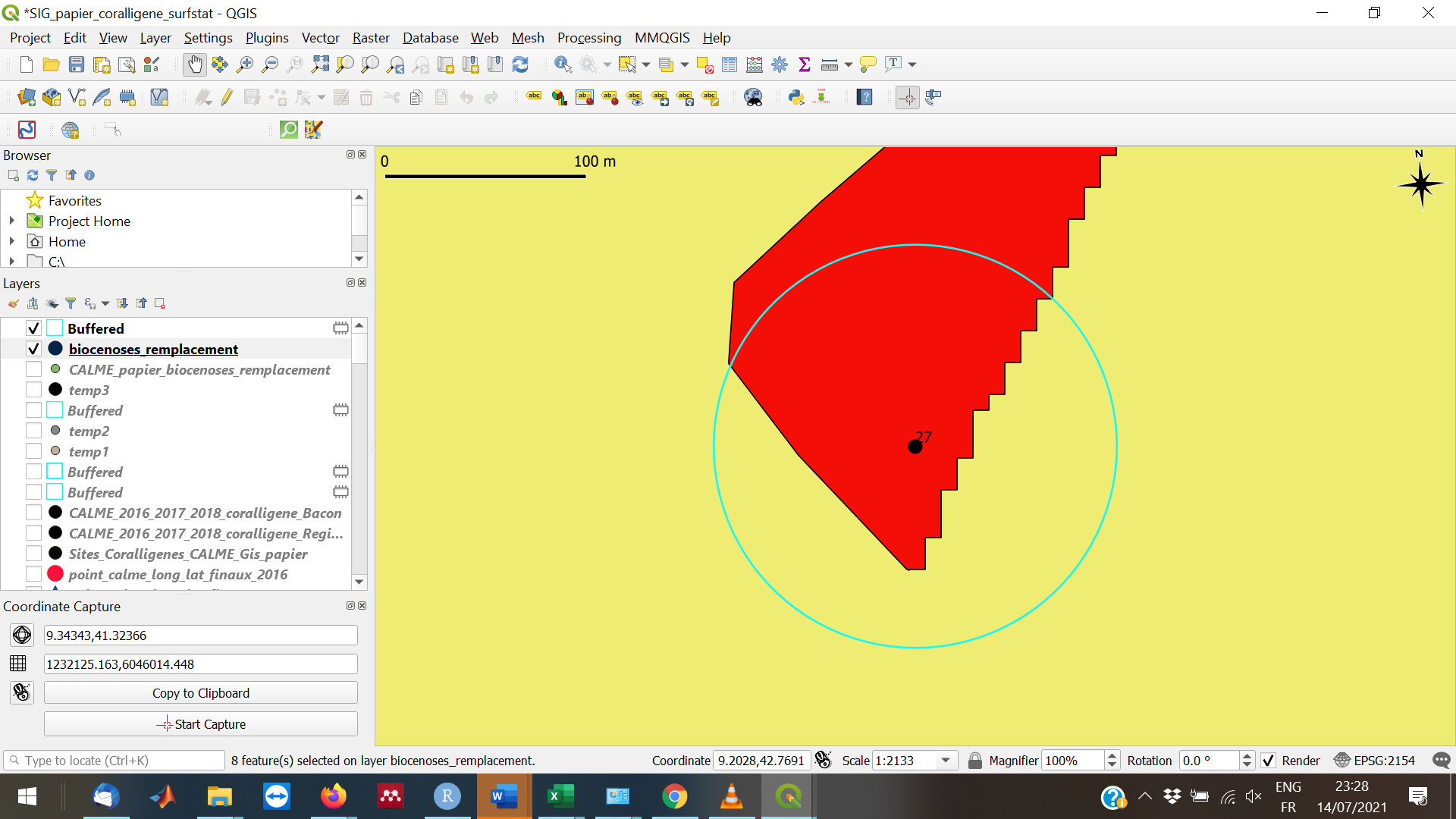


**Figure S6.** Biocenoses maps around the sampling sites. Blue circle indicating the 100 m radius around the recording position. Red = coralligenous reefs, yellow = sands, brown = rocky reef, green = *Posidonia oceanica*, grey = dead matte. Biocenoses maps obtained from the DONIA EXPERT program, https://medtrix.fr/portfolio_page/donia-expert/.

**
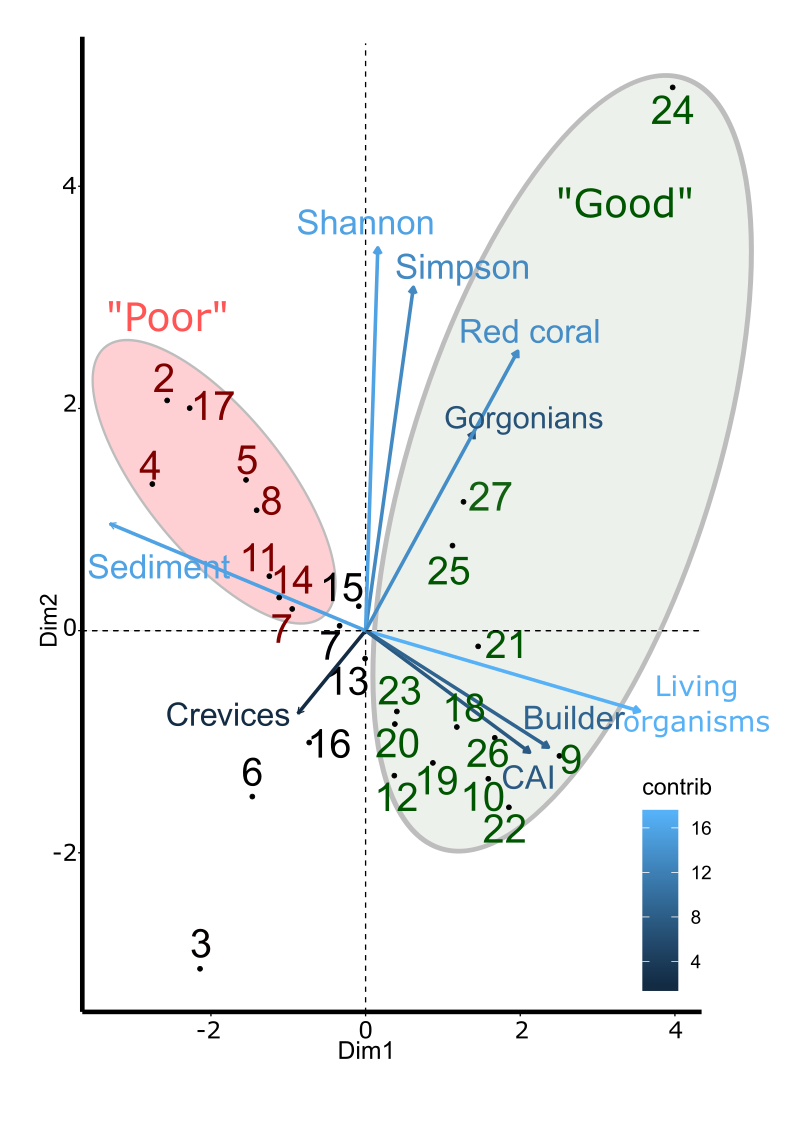
**

**Figure S7.** Principal component analysis (PCA) highlighting the relationships between habitat variables and the sites (numbers, *c.f.*, supplementary table S1) used to define environmental status categories. More relevant variables are light blue. The green ellipse contours the 13 sites associated to “good” environmental state, the red ellipse the 8 sites in less good (“poor”) condition, characterized by sediment (mud) and low levels of benthic assemblages. Six sites could not be clearly attributed to one of these two environmental statuses. Builder = Structuring species, Posidonia% = percent of *Posidonia oceanica*, Rock % = percent of rocky substrate, Coral %: percent of coralligenous outcrops.
